# A Passive Localization Algorithm That Integrates RSSI and PDOA Location Observations in a Mixed Scene

**DOI:** 10.3390/s26196314

**Published:** 2026-10-06

**Authors:** Gui Li, Mingxuan Chen, Heyang Zhang, Kairui Jin, Hanzhi Zhang, Xinrui Wang, Hang Zhou, Zhenyu Feng, Guangxing Li, Wenjun Chen, Jian Li, Yong Zhao

**Affiliations:** 1 The 10th Research Institute of China Electronics Technology Group Corporation, Chengdu 610036, China; 2 School of Communication & Information Engineering, University of Electronic Science and Technology of China, Chengdu 611731, China; 3 China Mobile Chengdu Institute of Research and Development, Chengdu 610213, China

**Keywords:** passive localization, RFID, carrier phase, RSSI, PDOA

## Abstract

Passive radio frequency identification (RFID) localization is attractive for low-cost indoor positioning, but mixed line-of-sight (LOS) and non-line-of-sight (NLOS) propagation causes both RSSI fluctuations and carrier-phase ambiguity. This study proposes a coarse-to-fine PRTFC–SD-PDOA framework in which an RSSI fingerprint likelihood defines the admissible decision domain, candidate-wise phase ambiguity is resolved inside that domain, and normalized RSSI and phase costs are jointly minimized. Dominant-reflection compensation is used when the reflecting surface is known, and temporal filtering is applied to repeated observations. In independently repeated simulations, the joint estimator achieved RMSEs of 0.098 m in LOS and 0.250 m in the mixed scene. In a nine-position R420 experiment, the particle-filtered estimator achieved mean point-wise RMSEs of 0.122 m in LOS and 0.457 m in the mixed scene; the corresponding Kalman-filtered results were 0.118 and 0.424 m. Thus, both filters reduced the variability of the unfiltered joint estimates, while the Kalman filter was slightly more accurate for the stationary, approximately Gaussian measurement sequences considered here.

## 1. Introduction

With the rapid development of the Internet of Things (IoT) and intelligent sensing technologies, indoor positioning has attracted extensive attention in both public and industrial applications [1,2]. In response, passive localization technologies have been widely investigated and continuously developed in recent years [3].

Among existing indoor localization technologies, ZigBee-based localization systems [4] are easy to deploy and cost-effective, but require a large number of nodes to improve communication efficiency. Ultra-wideband (UWB) technology [5] provides high positioning accuracy; however, it generally relies on line-of-sight propagation and involves high hardware and deployment costs. Wi-Fi-based localization methods [6] offer wide coverage but are susceptible to environmental interference and signal fluctuations. Infrared-based systems [7] are easily blocked by surrounding objects and are only suitable for limited scenarios. Compared with these indoor localization technologies, passive RFID systems [8,9] have attracted considerable attention because of their low cost, low power consumption, flexible deployment capability, and strong environmental adaptability.

RSSI-based localization methods have been widely investigated because of their low hardware complexity and simple implementation [10,11]. Representative systems such as SpotON and LANDMARC estimate target positions according to RSSI characteristics and reference-tag-assisted localization mechanisms [12,13]. More recent studies have incorporated anomaly compensation [14] or derived feasible tag regions from RSSI-indexed antenna sensitivity functions while explicitly accounting for measurement uncertainty [11].

In recent years, passive ultra-high frequency (UHF) RFID positioning technologies have attracted increasing attention. Early studies mainly focused on wireless channel modeling and signal propagation analysis for passive RFID systems. Griffin et al. established a backscatter link budget model for passive RFID systems, providing theoretical support for subsequent RFID channel analysis and compensation methods [15].

Mainstream RFID localization methods mainly include RSSI [16], angle of arrival (AOA) [17], time difference of arrival (TDOA) [18], and carrier-phase-based techniques [19,20,21]. AOA-based methods estimate positions using signal arrival angles, whereas TDOA-based methods rely on signal propagation time differences between receivers. Although these methods can improve positioning accuracy, they generally require complex antenna calibration, high synchronization accuracy, or high computational complexity [22,23,24].

In recent years, increasing attention has been paid to RFID localization methods based on carrier-phase information. Compared with RSSI observations, carrier-phase measurements provide fine-grained ranging information [19]. However, their accuracy can also be affected by the direction-dependent complex response of the tag and its at-tached object [20]. Existing studies have introduced phase information into fingerprint localization [25], particle-swarm-optimization-based PDOA localization [26]. More recently, RSSI and phase time-series have been combined with machine-learning models to improve robustness to industrial multipath fading, operational variability, and signal fragmentation [27].

Although existing phase-based localization methods have achieved promising performance, their robustness and positioning accuracy are still significantly affected by mixed line-of-sight (LOS) and non-line-of-sight (NLOS) propagation environments. To address this issue, this study proposes a passive RFID localization algorithm integrating RSSI and carrier-phase observations. In mixed LOS/NLOS environments, geometric reflection modeling is employed to transform NLOS propagation paths into equivalent LOS paths according to the spatial distribution of obstacles and antenna arrays. Subsequently, RSSI and carrier-phase observations are jointly fused for localization, while a particle filter is introduced to suppress observation noise and improve localization stability.

The closest antecedent is our earlier CMC study [28], which combined RSSI trilateration confidence regions, conventional SD-PDOA hyperbolic solutions, and an improved Kalman filter. The present study does not repeat that pipeline. It replaces trilateration with an interpolated PRTFC fingerprint likelihood, restricts the phase search to a connected high-likelihood component, resolves the integer ambiguity independently for every candidate and antenna pair, and minimizes a normalized joint RSSI-phase cost. It also isolates reflection compensation from fusion through controlled ablation and evaluates PF and KF with parameters fixed on calibration data.

Accordingly, the main contributions are a coarse-to-fine probabilistic formulation that uses RSSI as an admissible spatial domain rather than as a second coordinate estimate; candidate-wise ambiguity handling and normalized MAP fusion; an explicit image-antenna formulation for a known dominant reflector, with a reliability fallback when that geometry is unavailable; and component-level ablation, matched PF-KF comparison, runtime reporting, and nine-position R420 validation.

## 2. Methods

To address the limitations of existing passive RFID localization methods in mixed LOS/NLOS environments, this study proposes a joint RSSI–PDOA localization framework for passive indoor positioning. The proposed method first transforms NLOS propagation paths into equivalent LOS paths through geometric reflection modeling based on obstacle distributions and reflective surfaces. Subsequently, RSSI observations are employed for coarse localization, while SD-PDOA phase observations are utilized for fine-grained position estimation. Finally, a particle filter is introduced to suppress stochastic observation noise and improve localization stability.

### 2.1. Positioning Technology Based on RSSI

RSSI-based localization estimates the distance between the reader and the target tag according to signal attenuation characteristics. By combining the measured distances with geometric or fingerprint-based localization models, the target position can be determined. In fingerprint-based localization, an offline database is first established by mapping RSSI observations to spatial coordinates, and real-time RSSI measurements are subsequently matched with the predefined fingerprints during the online positioning stage.

The LANDMARC system improves localization accuracy by introducing reference tags with known coordinates. However, dense deployment of reference tags inevitably increases hardware cost and may introduce mutual interference in practical indoor environments. To address these limitations, the proposed Pseudo-Reference Tag Feature Comparison (PRTFC) algorithm expands the fingerprint space through spatial interpolation. By introducing pseudo-reference tags, the algorithm increases fingerprint density without requiring additional physical tags, thereby improving coarse localization performance.

**Figure 1:**
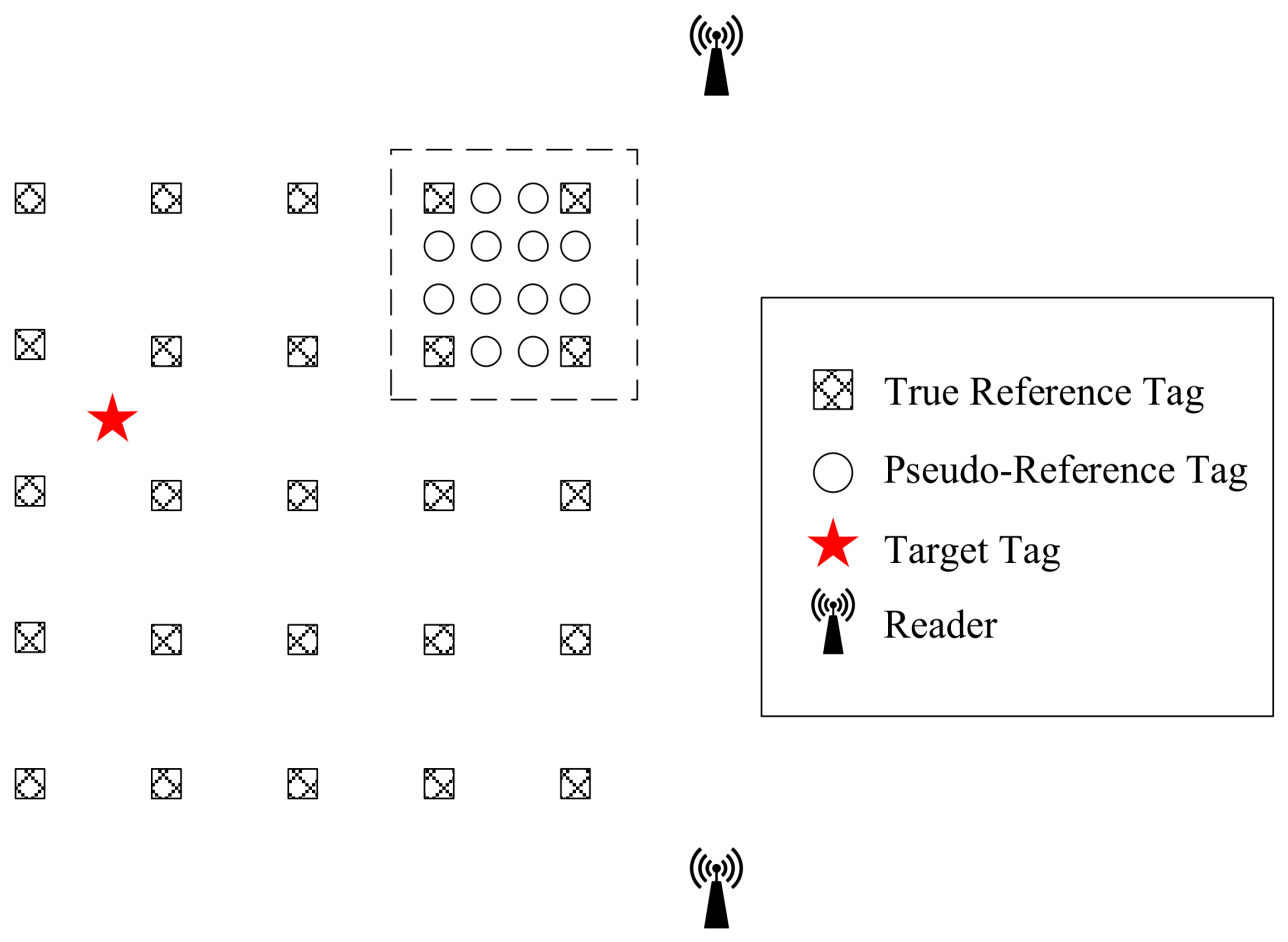
Schematic diagram of PRTFC positioning system. The dashed box highlights the interpolation of pseudo-reference tags between four neighboring real reference tags.

As illustrated in Figure 1, the PRTFC system arranges reference tags in a two-dimensional grid structure. Each subregion enclosed by four adjacent real reference tags is further divided into *N* × *N* cells of equal size. By applying linear interpolation to the RSSI observations of the neighboring reference tags, pseudo-reference tags can be generated to enhance the fingerprint resolution of the localization space.

The horizontal interpolation process is expressed as follows:

(1) $$\begin{array}{c} R_{k}(L_{p,b})=R_{k}\left(L_{a,b}\right)+p\times \frac{R_{k}\left(L_{a+N,b}\right)-R_{k}\left(L_{a,b}\right)}{N}\\ =\frac{p\times R_{k}\left(L_{a+N,b}\right)+\left(N-p\right)\times R_{k}\left(L_{a,b}\right)}{N} \end{array}$$

Similarly, the vertical interpolation process can be expressed as:

(2) $$\begin{array}{c} R_{k}(L_{a,q})=R_{k}\left(L_{a,b}\right)+q\times \frac{R_{k}\left(L_{a,b+N}\right)-R_{k}\left(L_{a,b}\right)}{N}\\ =\frac{q\times R_{k}\left(L_{a,b+N}\right)+\left(N-q\right)\times R_{k}\left(L_{a,b}\right)}{N} \end{array}$$

where

- *R_k_*(*L_i_*_,*j*_)—the RSSI of the reference tag in the i-th row and j-th column read by the k-th antenna;
- *a* = ⌊*i*/*N*⌋, *b* = ⌊*j*/*N*⌋;
- 0 ≤ *p* = *i*%*N* ≤ *N* − 1, 0 ≤ *q* = *j*%*N* ≤ *N* − 1.

Based on the above interpolation process, the fingerprint database of the positioning space can be established. During online localization, the RSSI fingerprint of the target tag is first acquired and subsequently matched with the constructed fingerprint database. Let the position fingerprint of the i-th tag be located be **Ω***_i_*, as shown in Equation (3).

**Ω***_i_* = (Ω*_i_*_1_,Ω*_i_*_2_,⋯,Ω*_in_*) (3)

where

- Ω*_ik_*—the RSSI value of the i-th tag to be located collected by the k-th reader.

**Figure 2:**
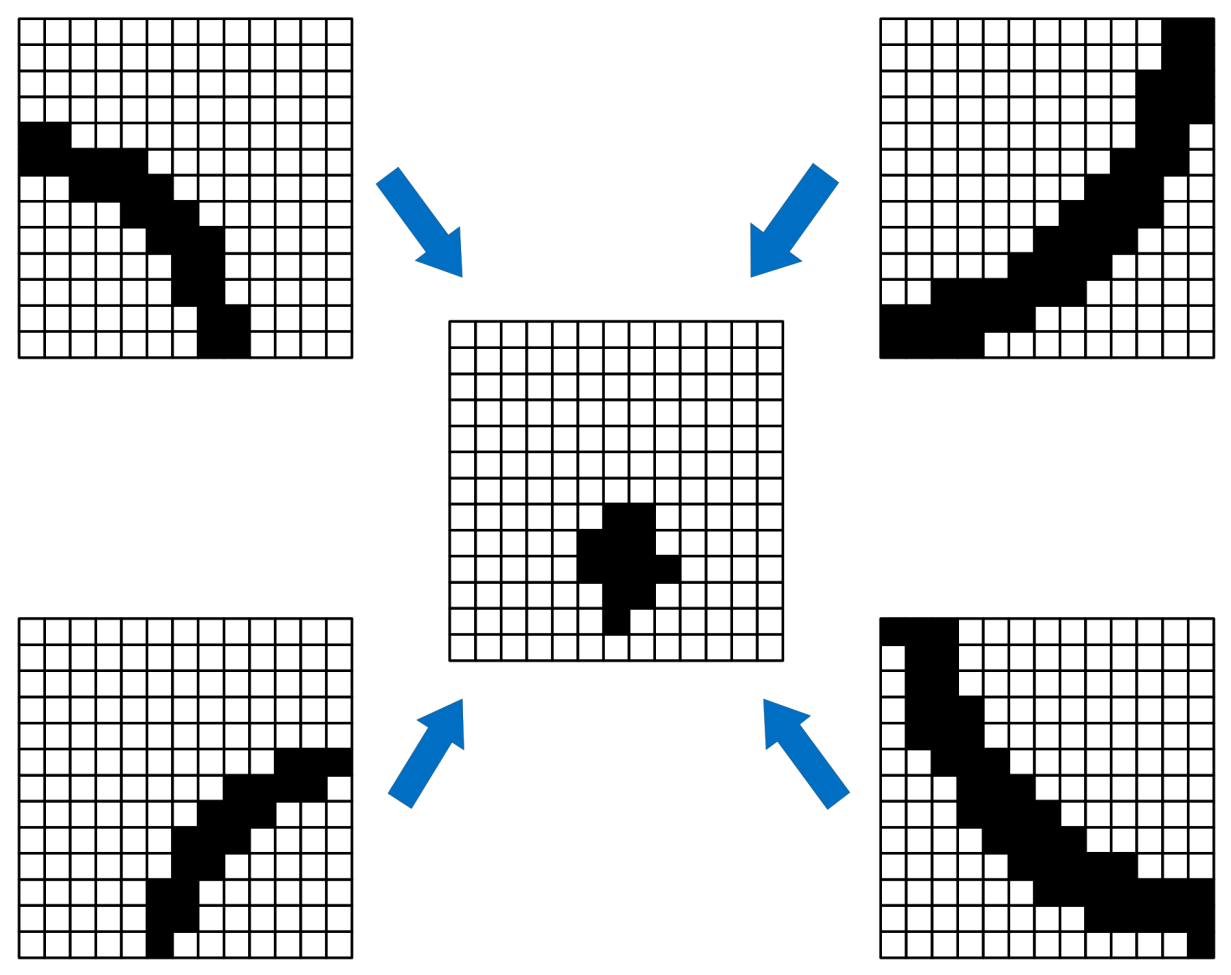
Probabilistic image formation process. The black cells denote candidate regions, and the blue arrows indicate their intersection.

To determine the position of the target tag, the algorithm first screens the nearest neighbor tags based on the RSSI values of the target tag and the reference tags associated with each antenna. The absolute difference between these RSSI values is calculated and substituted into a dynamic indication function to establish an adaptive threshold. Positions with absolute differences below the threshold are assigned a value of 1, whereas positions exceeding the threshold are assigned a value of 0. The positions assigned a value of 1 collectively form a probability image. Each antenna generates an individual probability image for the target tag. By intersecting these probability images, a final probability image is created, revealing the area where the target tag is most likely to be located, as illustrated in Figure 2.

The dynamic indication function adaptively adjusts the threshold according to the localization requirements, ensuring that the number of effective pixels in the final probability image remains within a specified range.

The specific procedure is summarized as follows:

1. Determine the Valid Pixel Interval: Establish an appropriate range for the number of valid pixels in the final probability image;
2. Calculate the initial threshold: compute the maximum absolute RSSI difference between the target tag and all reference tags, and use it as the upper bound of the initial threshold. The lower bound is initialized to 0, while the median value between the upper and lower bounds is selected as the initial threshold.

If the number of effective pixels falls within the predefined range, no further adjustment is required. Otherwise, if the number of valid pixels is below the lower bound, the current threshold is assigned as the new lower bound, and the threshold is recalculated using the updated bounds. Conversely, if the number of valid pixels exceeds the upper bound, the current threshold is assigned as the new upper bound, followed by threshold recalculation.

The above process is iteratively executed until the number of effective pixels satisfies the predefined condition. However, if the effective pixel interval is excessively narrow, the algorithm may fail to identify an appropriate threshold, potentially leading to an infinite loop. To avoid this issue, a maximum iteration limit is introduced. Once the number of iterations exceeds the specified limit, the threshold search process is automatically terminated.

### 2.2. Positioning Technology Based on PDOA

**Figure 3:**
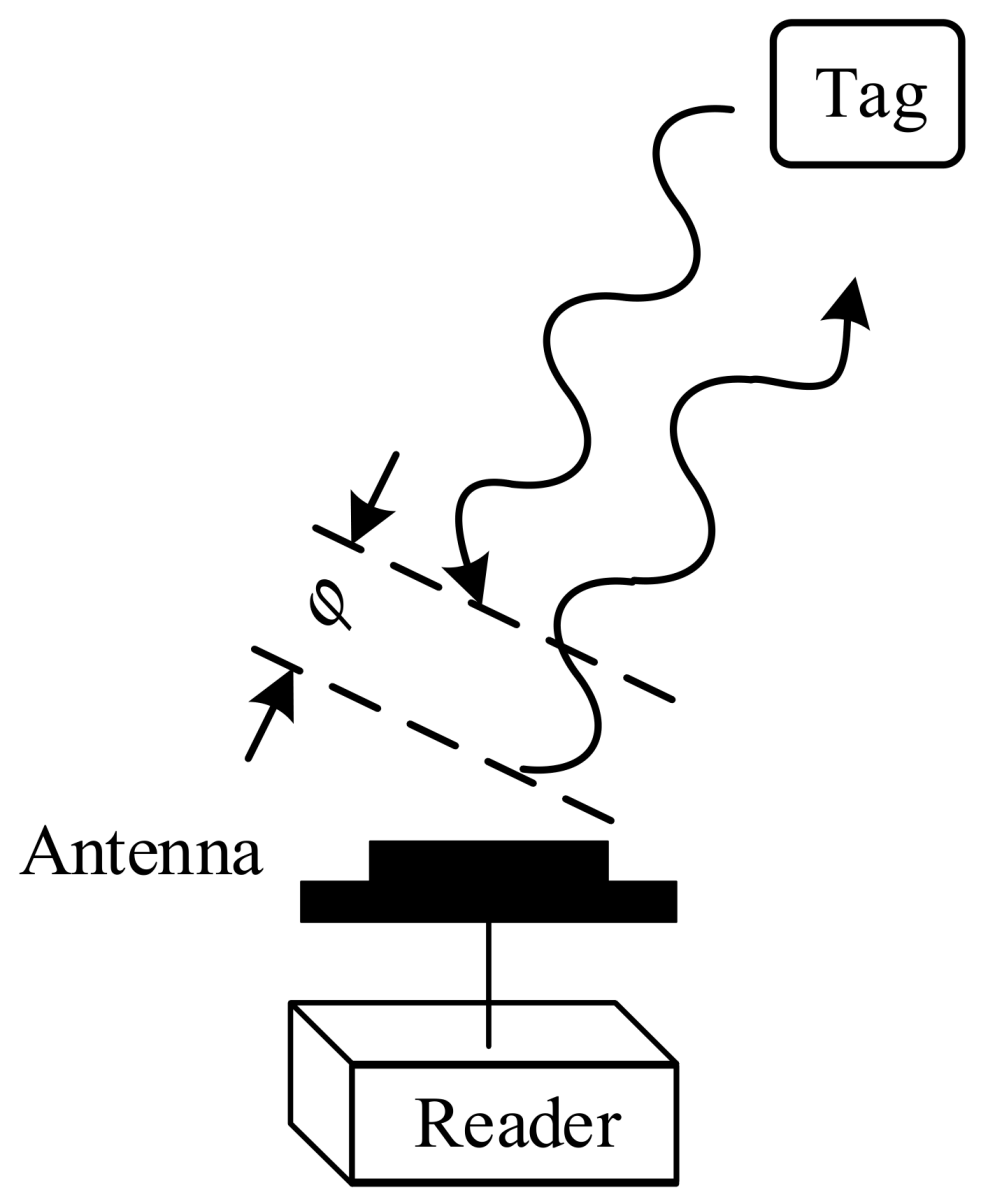
Schematic diagram of phase ranging principle. The arrows indicate the forward and backscattered signal propagation directions.

The methodology employing Phase Difference of Arrival (PDOA) technology leverages phase information to achieve precise positioning. In this framework, the RFID reader transmits radio frequency signals, which are subsequently modulated by passive tags to produce backscattered signals that return to the reader via the spatial channel. The time difference for the signal to traverse to the target tag and back is denoted as *τ*. The phase difference between the transmitted and received signals can be computed by multiplying the propagation time *τ* by the angular frequency *ω* of the signal. This phase difference facilitates the accurate determination of the distance between the reader and the tag. The principle of phase ranging is depicted in Figure 3.

Each time the phase of the signal changes by half a wavelength, a periodic transition occurs. The path length *R* of the straight-line propagation between the reader and the target tag is represented in Equation (4).

(4) $$R=\frac{\lambda }{2}(\frac{\varphi }{2\pi }+N)$$

where

- *λ*—signal wavelength;
- *φ*—the carrier phase value of the signal;
- *N*—the total number of cycles experienced.

The variation range of the phase is [0,2*π*]. If *R* exceeds *λ*/2, the number of complete periods experienced by the phase must be clearly defined to determine its value accurately. For high-frequency signals, the short wavelength makes direct phase-based ranging challenging. Therefore, phase ambiguity resolution is required, which can generally be classified into three domains: time domain, frequency domain, and spatial domain.

**Figure 4:**
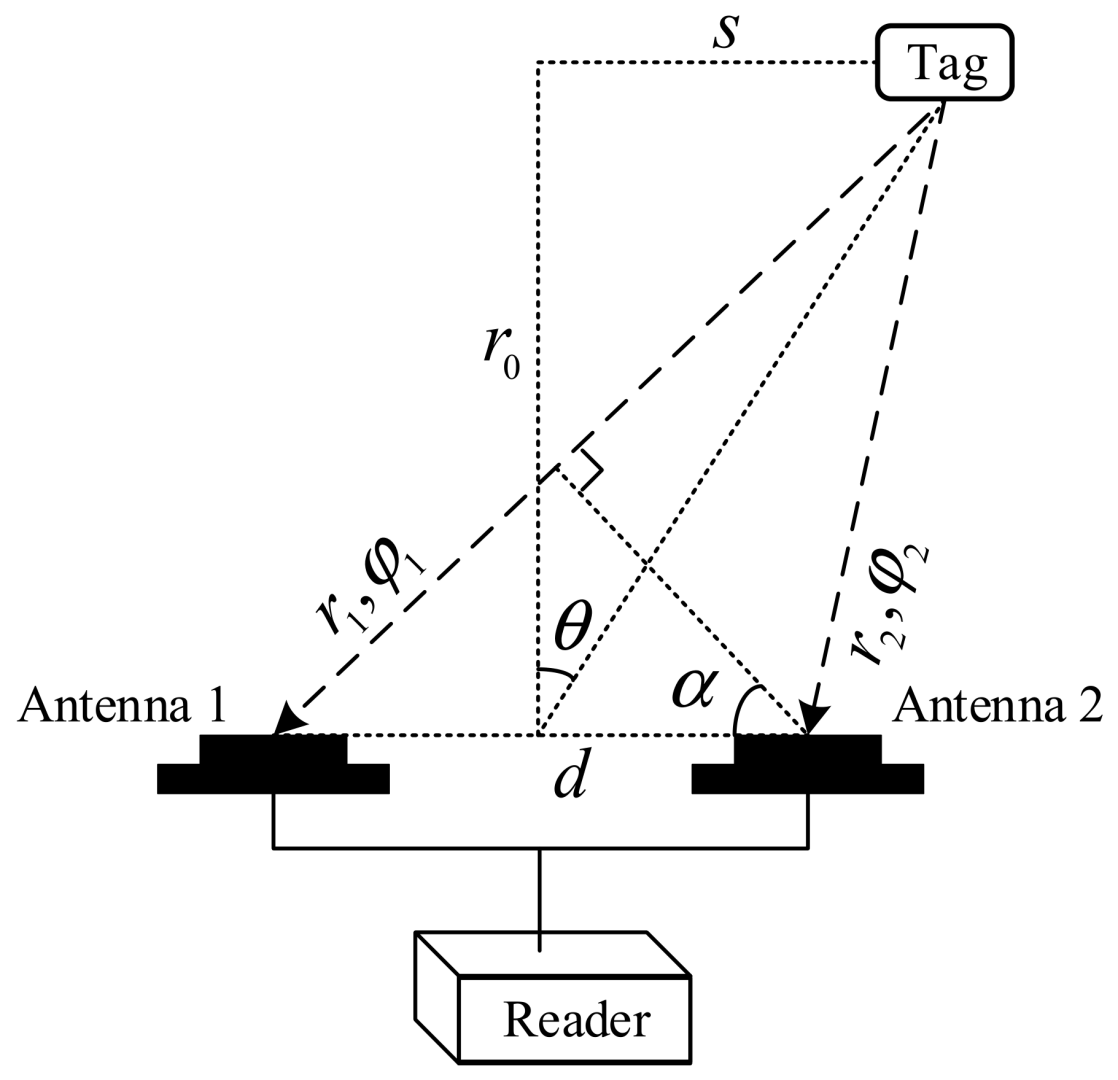
Schematic diagram of the positioning principle of the SD-PDOA algorithm.

In this study, we implement a positioning system based on Spatial Domain-Phase Difference of Arrival (SD-PDOA). This system is designed to collect phase data from tags through the utilization of two antennas operating at the same frequency, enabling the calculation of the carrier phase difference. This phase difference is subsequently transformed into a distance difference. By leveraging the hyperbolic properties, wherein the distance difference from any point on the hyperbola to the two foci remains constant, a hyperbolic model is established involving the tag and the antennas. The position of the tag can be accurately determined using two antenna groups, resulting in a total of four antennas. The schematic representation of the positioning principle of the SD-PDOA algorithm is illustrated in Figure 4.

In Figure 4, *r*_1_ and *r*_2_ respectively represent the distances between the target tag and the reader antennas 1 and 2, *r*_0_ denotes the vertical distance from the target tag to the plane where the antenna group is positioned, while *d* represents the distance between the antenna group, and *s* represents the distance between the target tag and the perpendicular bisector of the antenna group. If all the above parameters except *s* are known, the following values can be resolved:

From the positional relationship in Figure 4, the distance difference from the target tag to reader antennas 1 and 2 can be derived. When *r*_0_>>*d*, the values of *α* and *θ* are approximately equal, we obtain Equation (5):

*r*_1_ − *r*_2_ = *d*sin*α*≈*d*sin*θ* (5)

The value of *α* can be calculated based on the relationship between the phase difference and distance of the two antennas, as shown in Equation (6):

(6) $$\theta \approx \arcsin\left[\frac{c}{4\mathrm{\pi f}\cdot d}\times (\varphi_{1}-\varphi_{2})\right]$$

Given that *r*_0_ is known, s can be calculated by Equation (7):

*s* = *r*_0_tan*θ* (7)

To prevent integer ambiguity in calculations, this positioning system must meet specific conditions: the carrier phase propagation distance between Antenna 1 and Antenna 2 should be less than the wavelength. In this system, the signal propagation distance is twice the distance from the antenna to the tag. Let

2 ⋅ (*r*_1_ − *r*_2_) = 2*d*sin*α* ≤ *λ* (8)

Solving it, the distance between the two antennas must satisfy Equation (9):

(9) $$d\leq \frac{\lambda }{2\mathrm{sin\alpha}}$$

The half-wavelength condition is a geometric design condition, not a guarantee against all measured phase errors. At 920 MHz, the wavelength is approximately 0.326 m and the implemented within-pair spacing d = 0.16 m is slightly below half a wavelength (approximately 0.163 m). In the ideal single-path, noiseless model, the path-difference bound |r_2_ − r_1_| ≤ d < λ/2 places the spatial path difference inside one unambiguous interval. Phase noise, multipath, tag orientation, unequal reflection phases, and calibration error can nevertheless move a measured differential phase across a wrapping boundary or bias it within that interval. We therefore do not assume that half-wavelength spacing completely removes ambiguity in measured data.

In the proposed estimator, RSSI first limits the admissible candidate region. For every retained candidate and antenna pair, the algorithm then selects the integer multiple of 2π that minimizes the wrapped phase residual, as described in Section 2.5. The short spacing reduces the number of plausible ambiguity branches but also amplifies the effect of phase noise on the inferred distance difference. This trade-off motivates the use of the RSSI constraint and calibrated phase variances.

**Figure 5:**
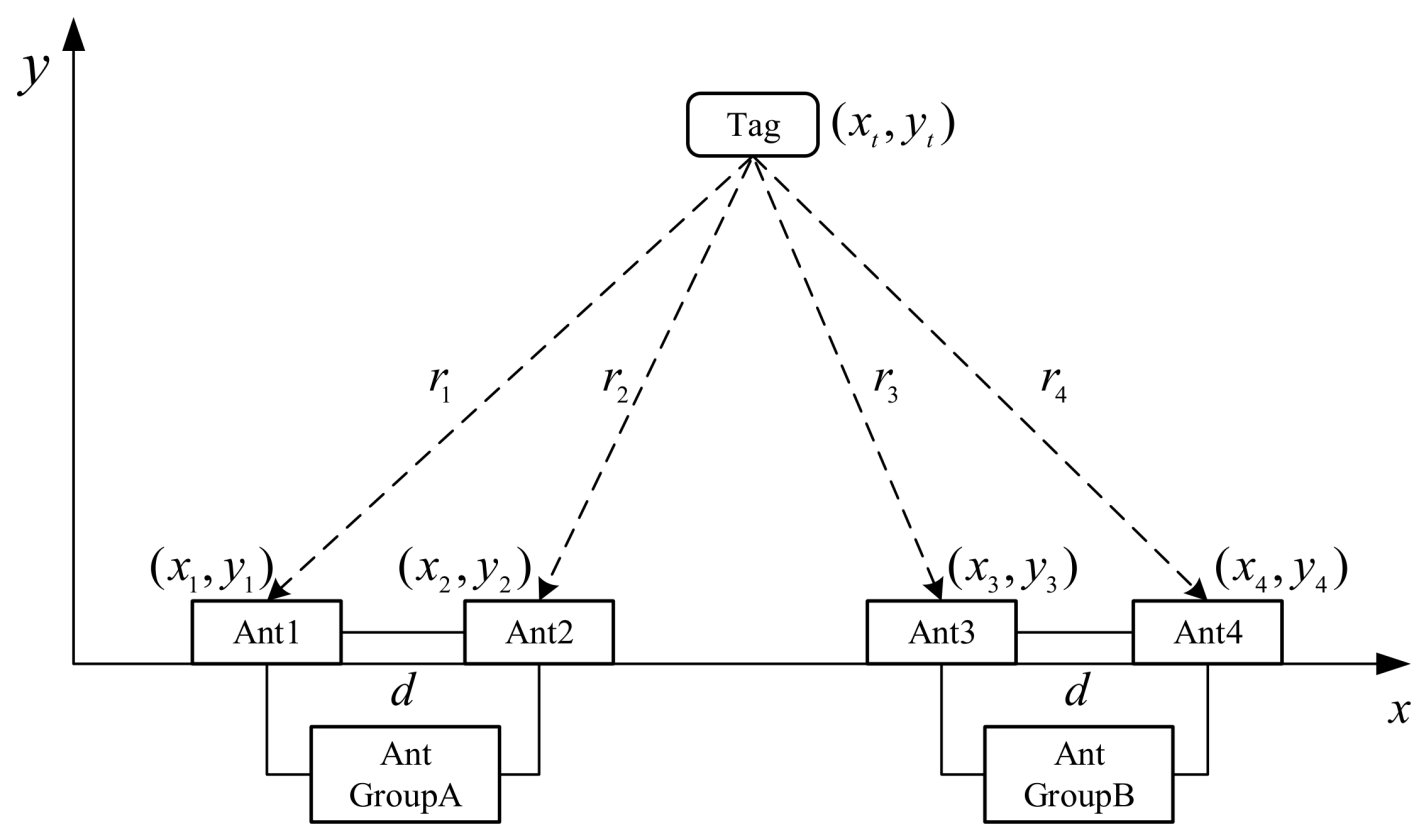
Schematic diagram of hyperbolic positioning of SD-PDOA.

Under these constraints, the SD-PDOA positioning system can incorporate two antenna groups to establish two hyperbolic models, enabling precise coordinate determination of the tags by solving the intersection points of the hyperbolic lines. As shown in Figure 5, in the indoor planar coordinate system, the tag coordinates are (*x_t_*,*y_t_*), and both the horizontal and vertical coordinates need to be measured. The coordinates of Antenna 1, 2, 3, and 4 are (*x*_1_,*y*_1_), (*x*_2_,*y*_2_), (*x*_3_,*y*_3_), and (*x*_4_,*y*_4_) respectively. In the actual positioning system, the specific coordinates of the four antennas are all known, allowing for the calculation of distances between each antenna and the tag using Equation (10):

(10) $$\begin{array}{l} r_{1}=\sqrt{{\left(x_{t}-x_{1}\right)}^{2}+{\left(y_{t}-y_{1}\right)}^{2}}\\ r_{2}=\sqrt{{\left(x_{t}-x_{2}\right)}^{2}+{\left(y_{t}-y_{2}\right)}^{2}}\\ r_{3}=\sqrt{{\left(x_{t}-x_{3}\right)}^{2}+{\left(y_{t}-y_{3}\right)}^{2}}\\ r_{4}=\sqrt{{\left(x_{t}-x_{4}\right)}^{2}+{\left(y_{t}-y_{4}\right)}^{2}} \end{array}$$

Within the same antenna group, the distance difference between the target tag and the two antennas can be obtained based on the phase difference. Given the position coordinates of each antenna, the hyperbolic equation system, as shown in Equation (11) can be solved:

(11) $$\left\{\begin{array}{l} \frac{{\left(x_{t}-x_{A}\right)}^{2}}{{a_{A}}^{2}}-\frac{{y_{t}}^{2}}{{b_{A}}^{2}}=1\\ \frac{{\left(x_{t}-x_{B}\right)}^{2}}{{a_{B}}^{2}}-\frac{{y_{t}}^{2}}{{b_{B}}^{2}}=1 \end{array}\right.$$

where

- ${a_{A}}^{2}={\left(\frac{r_{1}-r_{2}}{2}\right)}^{2}$, ${b_{A}}^{2}=\frac{d^{2}}{4}-{a_{A}}^{2}$;
- ${a_{B}}^{2}={\left(\frac{r_{3}-r_{4}}{2}\right)}^{2}$, ${b_{B}}^{2}=\frac{d^{2}}{4}-{a_{B}}^{2}$;
- $x_{A}=\frac{x_{1}+x_{2}}{2}$, $x_{B}=\frac{x_{3}+x_{4}}{2}$.

The antenna group in this positioning system is deployed along the *X*-axis, with 0 as the vertical coordinate and *x_A_* and *x_B_* as the horizontal coordinates, respectively. The solution set of hyperbolas typically comprises four components. Quadrants and regions are selected based on specific circumstances to retain reasonable positioning coordinates.

### 2.3. Particle Filter

The accuracy of the hyperbolic positioning method predicated on Spatial Domain-Phase Difference of Arrival (SD-PDOA) is inherently limited by the positioning model employed. To enhance positioning accuracy, we incorporate the particle filter algorithm to filter and estimate values derived from multiple positioning outcomes. The Particle Filter (PF) is a sequential Monte Carlo method that facilitates recursive Bayesian estimation through Monte Carlo simulation techniques. Its fundamental principle involves the utilization of weighted random samples to represent the posterior probability density of the random variables within the system, thereby enabling the estimation of the state value.

The coordinate state space vector model of the particle filter positioning system is defined as $X={\left[\begin{array}{cc} x & y \end{array}\right]}^{T}$, where x and y are the horizontal and vertical coordinates of the position of the RFID tag to be measured respectively. For a stationary RFID tag, the state transition matrix remains constant over time. Thus, the state transition function from state **X***_t_*_−1_ at time *t* − 1 and the current state **X***_t_* at time *t* can be expressed as:

**X***_t_* = **X***_t_*_−1_ (12)

The tag state at time t is iteratively predicted through the state transition equation, and then corrected based on the measured values input at time t. The initial coordinates of the tag to be tested are derived by establishing a hyperbolic equation for the link distance. The preliminary result of the SD-PDOA hyperbolic positioning algorithm is input into the system as the initial values *x*_0_ and *y*_0_ of the coordinates. The observation equation of the system as a whole can be expressed as:

**Z***_t_* = **HX***_t_* + **E***_t_* (13)

where

- **E***_t_*—the observation noise matrix at time t.

To sum up, based on the RFID tag positioning information collected at different times as the filtering input, the PF algorithm process is divided into the following steps:

1. Set the initial particle distribution: Assume that the initial state distribution follows *μ_j_* multi-dimensional standard normal distribution with a mean of a and a variance of *σ_j_*. The initial *K* particles and their weights are set as:

(14) $$\left\{\begin{array}{l} {\left\{X_{0}^{\left(i\right)}\right\}}_{i=1}^{K}∼N(\mu_{1},\mu_{2},\cdots ,\mu_{j};\sigma_{1},\sigma_{2},\cdots ,\sigma_{j}),j=1,2,\cdots ,M\\ {\left\{w_{0}^{\left(i\right)}\right\}}_{i=1}^{K}=\frac{1}{K} \end{array}\right.$$

where

- *M*—the total number of evaluated target tag positions.

2. Prediction: Random particles at the moment can be generated through the initial particle distribution and the above Equation (12):

(15) $$X_{t}^{\left(i\right)}=X_{t-1}^{\left(i\right)}+Q_{t}^{\left(i\right)}$$

where

- $Q_{t}^{\left(i\right)}$—the sampling noise matrix at time t.

3. Update correction: By obtaining the observed value **Z***_t_* at the t iteration moment, the particle weights at the current moment t are updated and corrected:

(16) $$w_{\iota }^{\left(i\right)}=w_{\iota -1}^{\left(i\right)}f_{R_{t}}(Z_{\iota }-H(X_{\iota }^{\left(i\right)}))$$

where

- **R***_t_*—error covariance matrix.

Then there is:

(17) $$f_{R_{t}}(Z_{\iota }-H(X_{\iota }^{\left(i\right)}))=\frac{1}{\sqrt{2\pi R_{t}}}e^{-\frac{{\left(Z_{\iota }-H(X_{\iota }^{\left(i\right)})\right)}^{2}}{2R_{t}}}$$

Normalizing the weights at the current time t results in:

(18) $${\tilde{w}}_{t}^{\left(i\right)}=\frac{w_{t}^{\left(i\right)}}{\sum_{i=1}^{k}w_{t}^{\left(i\right)}}$$

By using the particles and update weights at the current moment t, the filtering estimated value at the current iteration moment can be obtained:

(19) $${\hat{X}}_{t}=\sum_{i=1}^{\kappa }({\tilde{w}}_{\iota }^{\left(i\right)}X_{t}^{\left(i\right)})$$

4. Resampling stage: The size of the particle weight is used to determine whether it is retained, and an acceptance interval is set to evaluate whether the particle falls within this interval. In order to maintain the stability of the particle quantity, the particles with higher weights are replicated to fill the vacant positions, and all the reorganized particles are input into the state transition function to ensure the accuracy of the final positioning.
5. The loop executes steps 1 to 4 until the iteration termination condition is met to obtain the optimal solution for positioning.

Particle filtering represents the posterior distribution with weighted samples and can accommodate non-Gaussian or multimodal state distributions. Its computational complexity is O(KT), where K is the number of particles and T is the number of observations. For comparison, a two-dimensional random-walk Kalman filter (KF) was evaluated using the same joint-location sequence. The PF used 500 particles; process, measurement, and initialization standard deviations of 0.01, 0.30, and 0.30 m, respectively; and systematic resampling when the effective sample size fell below 0.5 K. The KF process and measurement variances were 1 × 10^−5^ and 0.0225 m^2^. These parameters were fixed using calibration sequences rather than the reported test coordinates. In the simulation comparison, each filter processed 1000 observations per location, and observations 201–1000 were used after burn-in.

### 2.4. Non-Line-of-Sight Path Transformation

**Figure 6:**
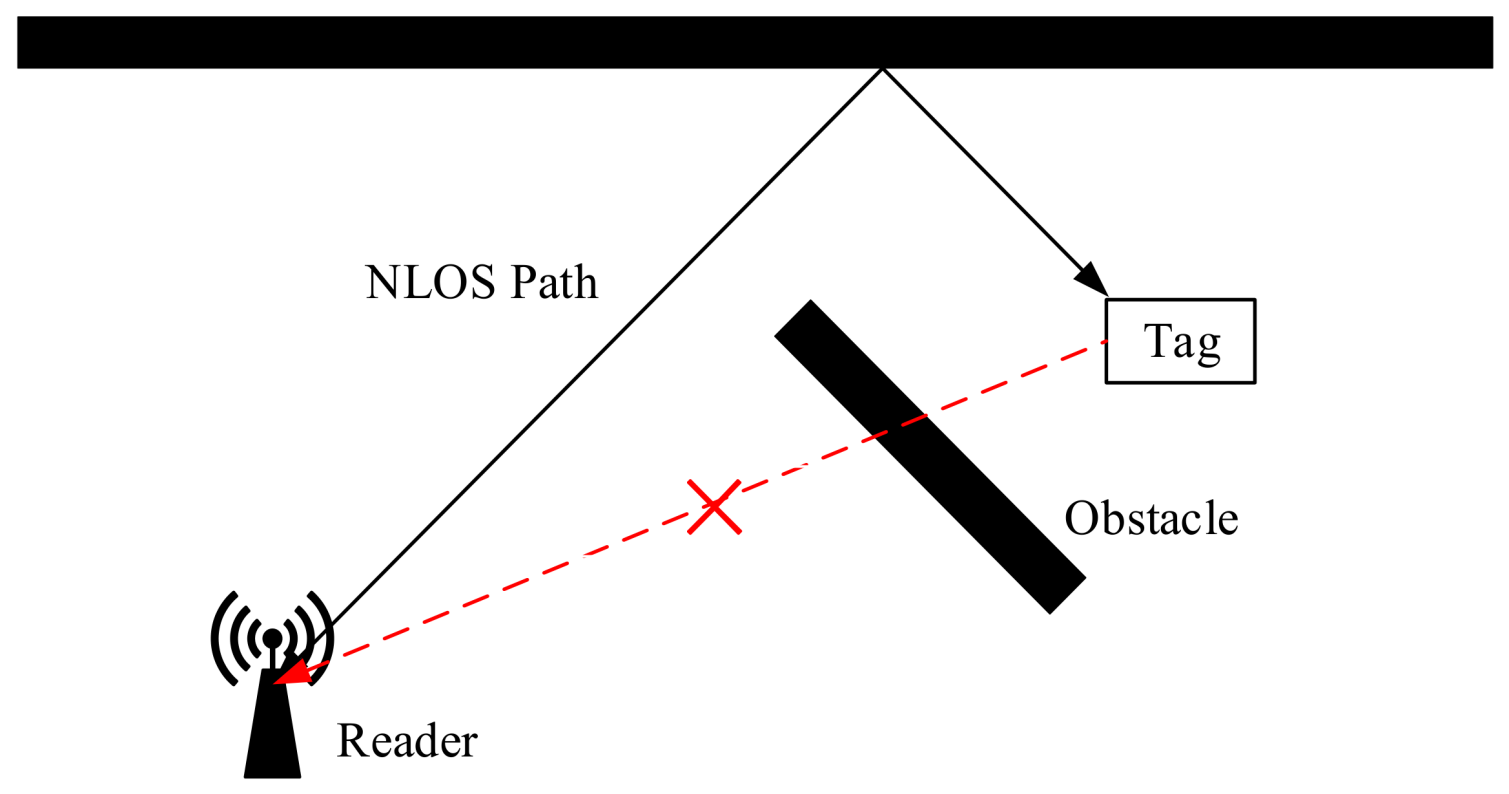
Schematic diagram of signal non-line-of-sight propagation. The arrows indicate the reflected signal path, and the red cross marks the blocked direct path.

In densely obstructed indoor environments, obstacles frequently block direct communication between RFID readers and target tags, causing line-of-sight (LOS) and non-line-of-sight (NLOS) signals to coexist. NLOS propagation (illustrated in Figure 6) induces substantial distance measurement errors that severely degrade overall system performance.

**Figure 7:**
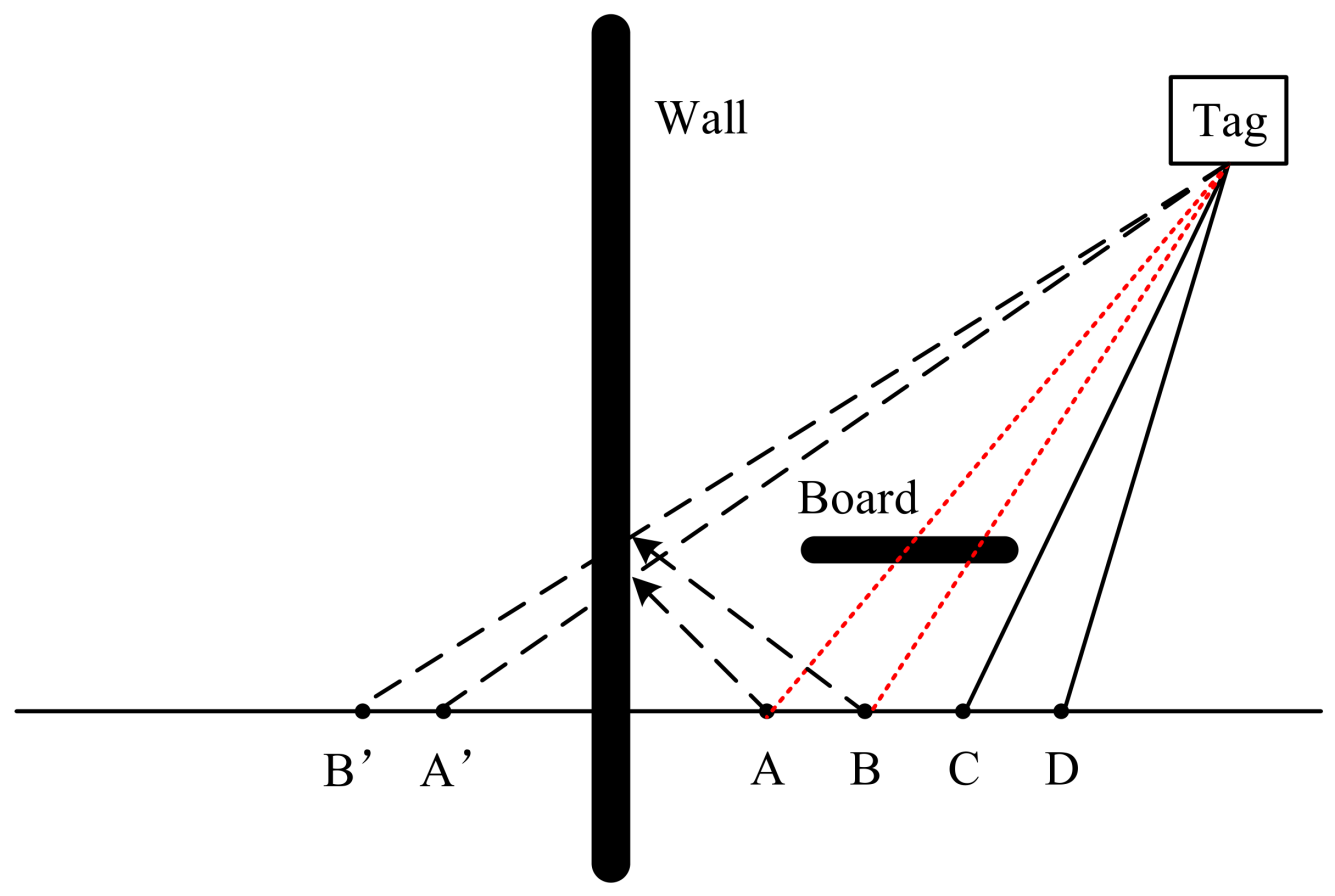
Schematic diagram of line-of-sight and non-line-of-sight positioning signal propagation. The red dashed lines indicate the direct paths blocked by the board.

**Figure 8:**
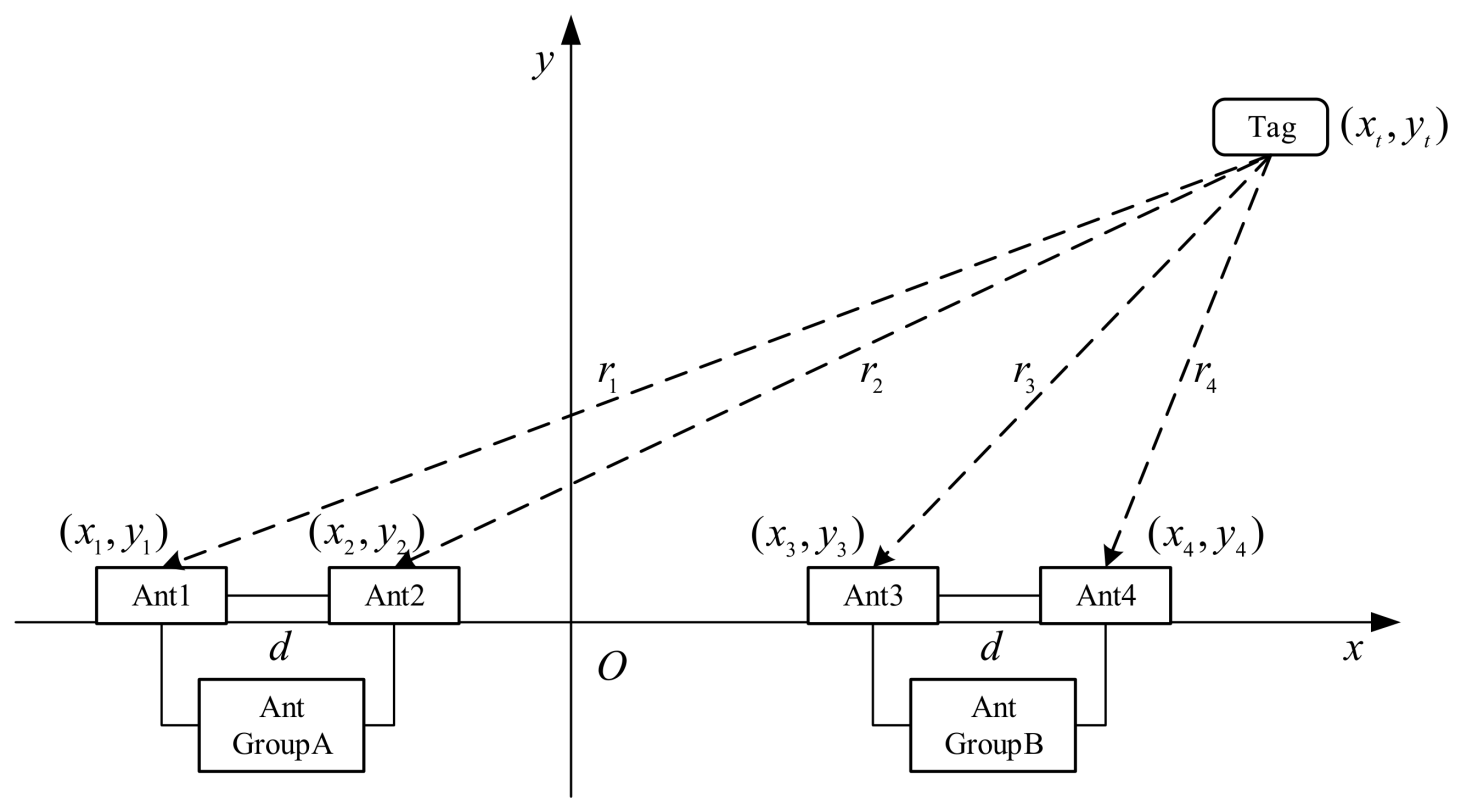
Schematic diagram of hyperbolic positioning of LOS and NLOS SD-PDOA.

For a dominant planar reflector represented in two dimensions by ax + by + c = 0, a physical antenna a_i = (x_i, y_i) is replaced by its image a_i′ = a_i − 2(ax_i + by_i + c)(a, b)/(a^2^ + b^2^). The length of the corresponding specular broken path equals the Euclidean distance from the tag candidate p to a_i′. For the passive backscatter round trip, the predicted propagation phase is therefore proportional to (4π/λ)||p − a_i′||. In the controlled mixed-scene simulation, the wall is x = 0, so the first antenna pair is mapped from (x_i, y_i) to (−x_i, y_i). The corresponding propagation geometry is shown in Figure 7. Figure 8 illustrates hyperbolic positioning using LOS and NLOS SD-PDOA.

For antennas i and j, the predicted differential phase contains the image-path term (4π/λ)(||p − a_j′|| − ||p − a_i′||), the calibrated antenna-bias difference, and any link-specific reflection-phase difference. In the simulation, a common reflection phase of π is applied to both antennas of the NLOS pair and cancels in the pairwise difference. In the R420 measurements, antenna-specific circular offsets were removed using calibration records. If a link-specific reflection phase or reflector geometry cannot be calibrated, the affected pair is assigned low or zero reliability instead of estimating that quantity from the test coordinate.

No explicit reflection-coefficient or incidence-angle correction is imposed by the present estimator. The RSSI branch is fingerprint-based, so repeatable site-specific attenuation present during calibration is represented by the fingerprint mean; the simulation models additional mixed-scene variability by increasing the RSSI noise standard deviation from 0.2 to 0.5 dB. The required prior information is therefore the antenna coordinates, the RSSI fingerprint map, and the line of a known dominant reflector for any phase pair that is geometrically compensated. Multiple, curved, moving, or unknown reflectors lie outside this single-dominant-path model; when no reliable dominant path is available, the corresponding phase observation must be down-weighted or excluded.

### 2.5. Proposed PRTFC-SDPDOA Algorithm

Although the final PRTFC probability image generally contains the target, a hard rectangular decision window may also retain locations with weak RSSI support. We therefore express the RSSI fingerprint mismatch as a normalized negative log-likelihood and retain the connected high-likelihood component containing the minimum-cost pixel. Carrier phase then supplies the fine spatial discrimination that RSSI alone cannot provide.

**Figure 9:**
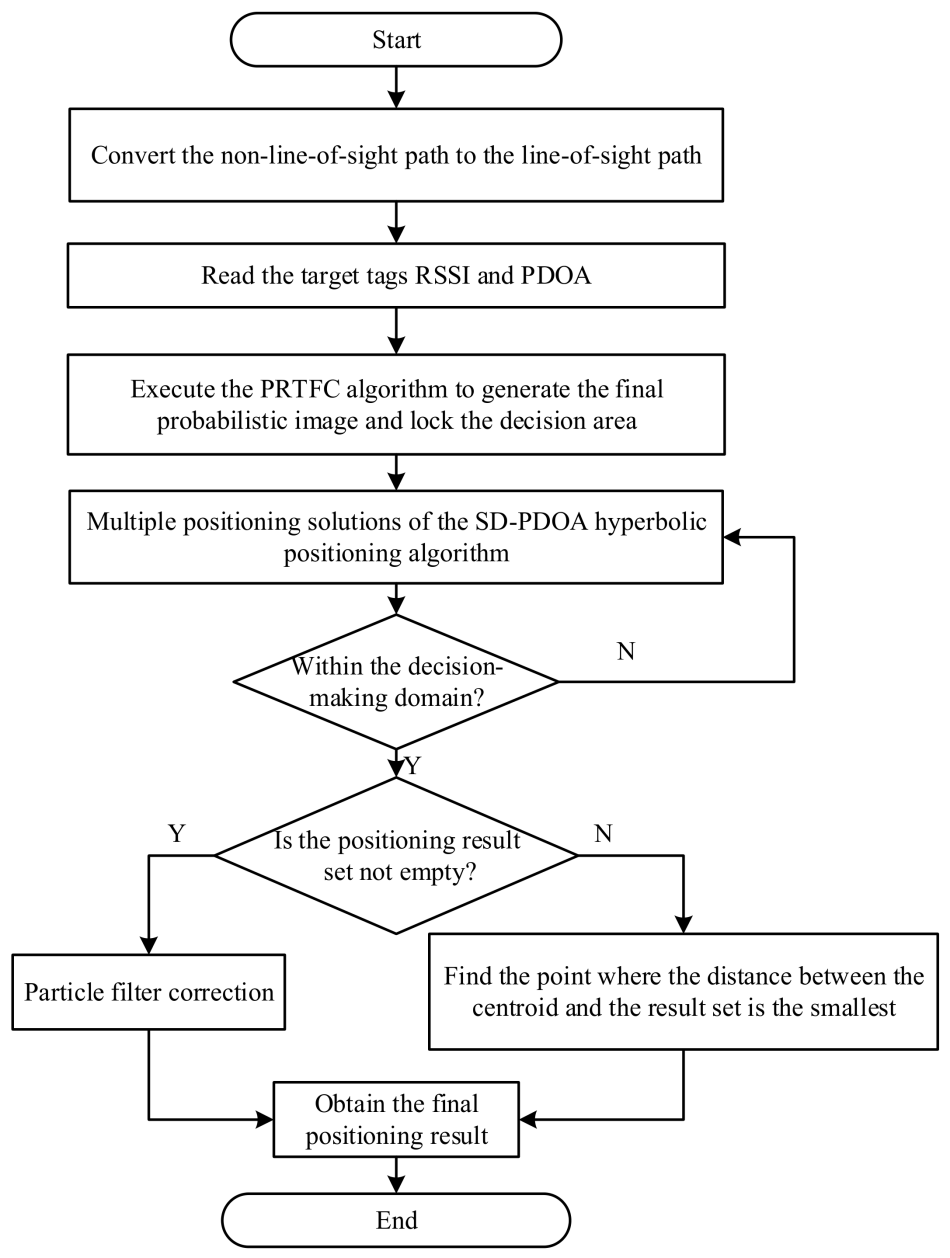
Flowchart of the joint positioning algorithm based on SD-PDOA and PRTFC.

For every candidate and phase–antenna pair, the integer ambiguity is selected by rounding the difference between the predicted and measured phase to the nearest multiple of 2π. The final estimate minimizes the sum of the normalized RSSI and phase costs within the retained RSSI component. The phase weight was set to 1 and the RSSI support threshold to 8 normalized negative-log-likelihood units using calibration positions excluded from the final test set. The overall workflow is illustrated in Figure 9.

The specific execution process is as follows.

Step 1: collect synchronized four-channel RSSI and four-channel carrier-phase observations from the target tag;

Step 2: when a dominant reflecting surface is known, transform the corresponding NLOS phase–antenna pair into an equivalent image-antenna geometry;

Step 3: calculate the normalized RSSI fingerprint cost on the candidate grid and retain the connected component satisfying the calibrated likelihood threshold;

Step 4: for every retained candidate, resolve each pairwise phase ambiguity independently and calculate the normalized SD-PDOA residual;

Step 5: add the normalized RSSI and phase costs and select the minimum-cost candidate as the unfiltered joint estimate;

Step 6: apply PF to repeated joint estimates; a KF with independently fixed parameters is evaluated as a comparator for stationary sequences;

Step 7: output the filtered position estimate.

## 3. Results

Throughout this section, the positioning error for each observation is defined as the Euclidean distance between the estimated and surveyed coordinates. RMSE is the square root of the mean squared positioning error over N observations. Lower RMSE therefore denotes better positioning performance; positioning accuracy is not treated as a separate numerical metric.

### 3.1. Simulation Analysis

**Figure 10:**
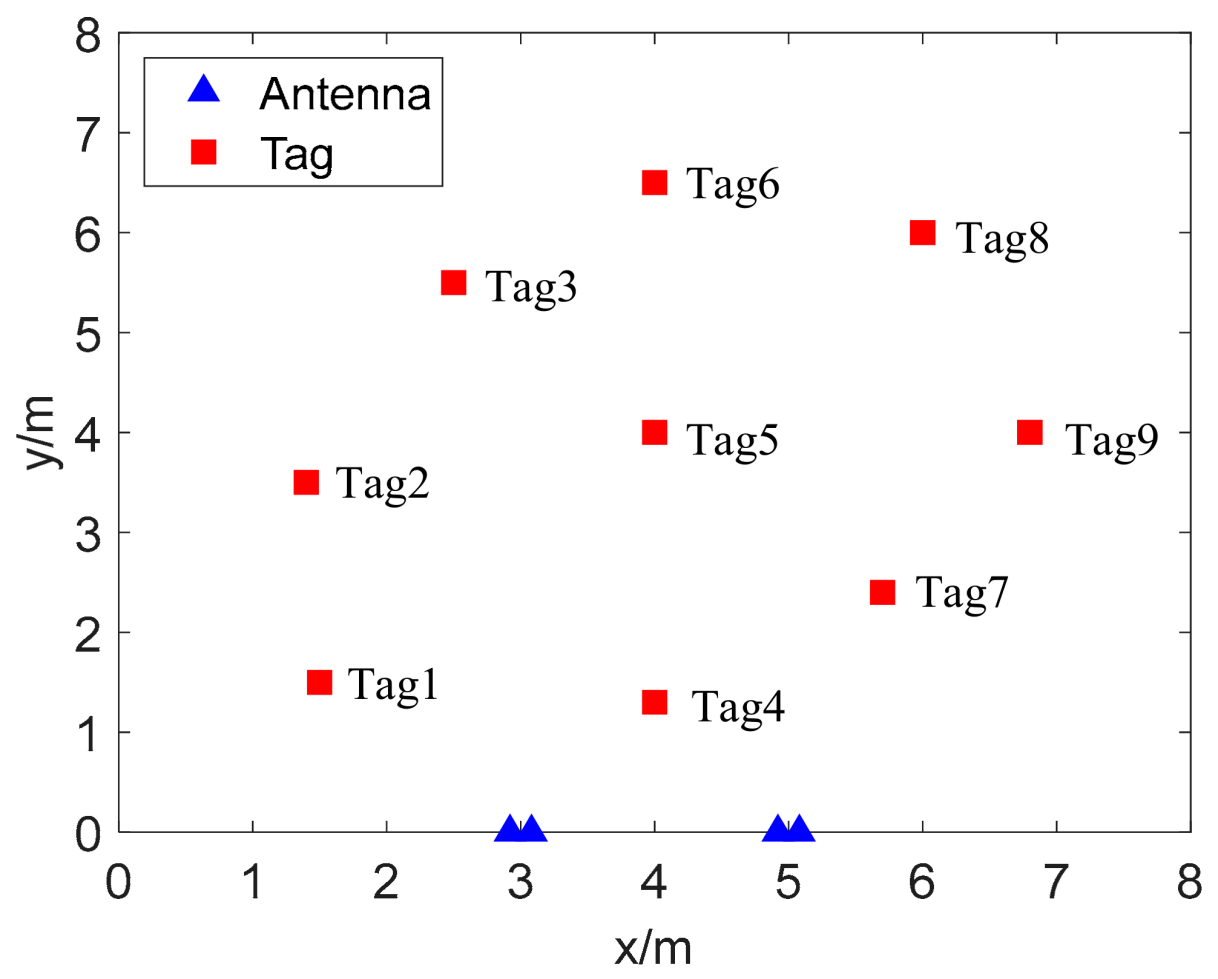
Positioning system deployment diagram.

The audited simulation used an 8 × 8 m area. RSSI antennas were located at (0, 0), (0, 8), (8, 0), and (8, 8) m, and phase antennas at (2.92, 0), (3.08, 0), (4.92, 0), and (5.08, 0) m. The carrier frequency was 920 MHz and the within-pair phase-antenna spacing was 0.16 m. Sixteen reference tags occupied all combinations of x, y ∈ {1, 3, 5, 7} m, and bilinear interpolation produced a 0.5 m RSSI fingerprint grid. The nine independent test positions were (1.5, 1.5), (1.4, 3.5), (2.5, 5.5), (4.0, 1.3), (4.0, 4.0), (4.0, 6.5), (5.7, 2.4), (6.0, 6.0), and (6.8, 4.0) m. Transmit power and path-loss exponent were 20 dBm and 2.3. RSSI and single-antenna phase-noise standard deviations were 0.2 dB and 0.1 rad in LOS, and 0.5 dB and 0.3 rad in the mixed scene. Sixteen calibration positions, distinct from the nine test positions, supplied 50 observations per scenario for parameter selection; final evaluation used 1000 observations per test position. Fingerprint, calibration, and test random seeds were 20260921, 20260923, and 20260924. No test-coordinate ground truth was used for phase ambiguity selection or estimator initialization. The deployment is shown in Figure 10.

**Figure 11:**
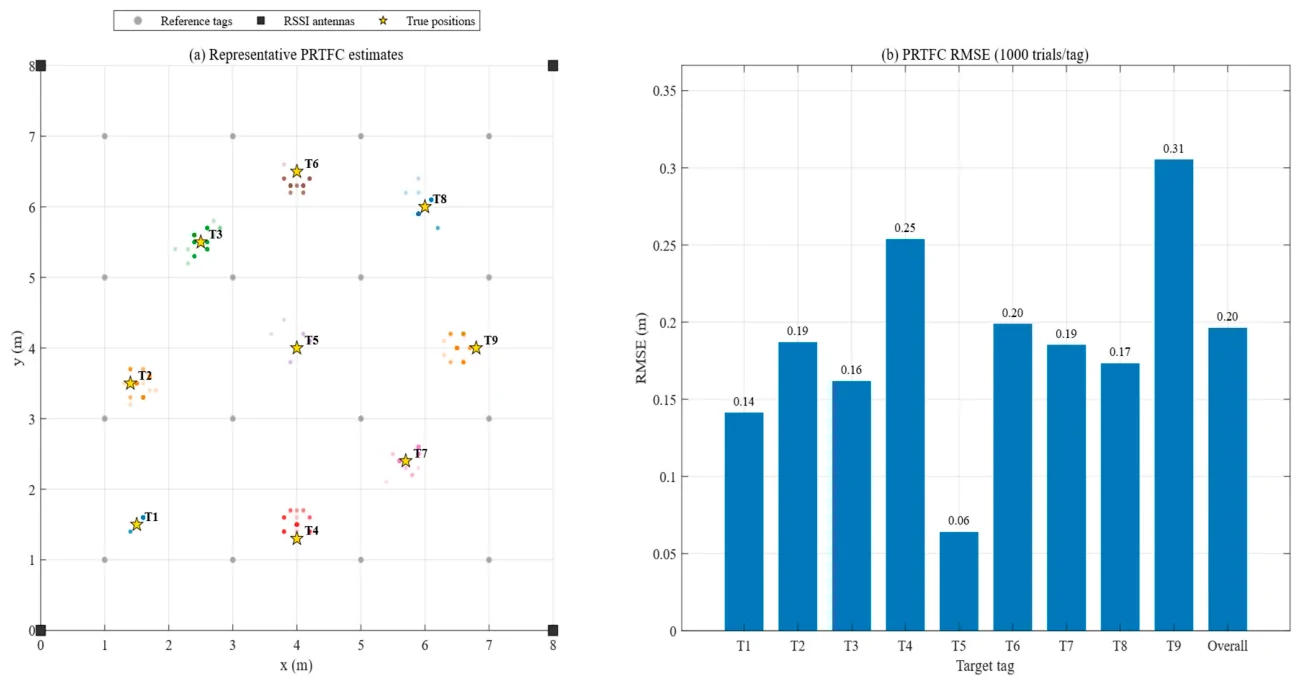
LOS performance of the PRTFC stage: (**a**) 100 representative estimates at each of nine target positions; (**b**) per-tag and overall RMSE calculated from 1000 observations per tag. In (a), the colored dots represent PRTFC position estimates, with different colors corresponding to different target tags.

Figure 11 summarizes the LOS performance of the PRTFC coarse-localization stage. The per-tag RMSE ranged from 0.064 to 0.305 m, and the overall RMSE over 1000 independent observations per tag was 0.196 m. The largest error occurred at Tag 9 near the boundary of the fingerprint region. PRTFC therefore provides a stable coarse estimate, although its accuracy remains position dependent.

**Figure 12:**
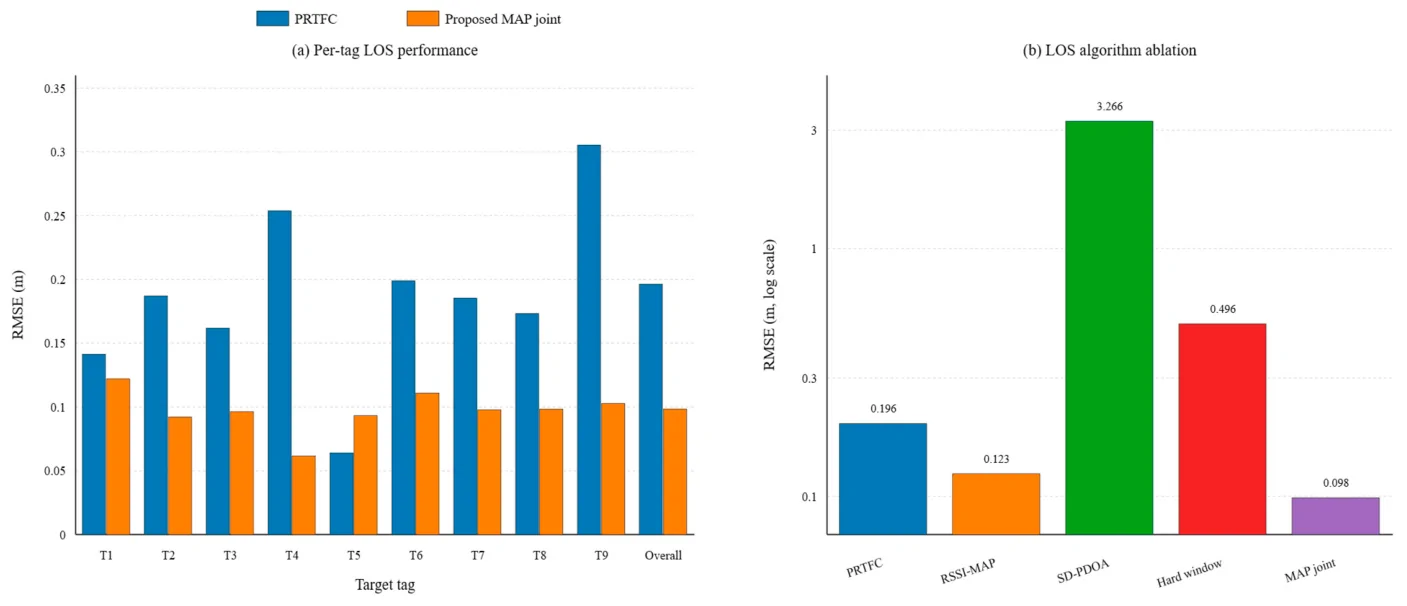
LOS localization results: (**a**) per-tag comparison of PRTFC and the proposed probabilistic MAP joint estimator; (**b**) overall ablation of PRTFC, dense RSSI-MAP, standalone SD-PDOA, the original hard-window joint method, and the proposed method. The logarithmic ordinate in (**b**) displays the large range of phase-only errors.

Figure 12 compares the LOS estimators. The overall RMSEs of PRTFC, dense RSSI-MAP, standalone SD-PDOA, the original hard-window joint method, and the proposed probabilistic MAP joint method were 0.196, 0.123, 3.266, 0.496, and 0.098 m, respectively. The proposed method reduced RMSE by 49.8% relative to PRTFC, by 80.2% relative to the original hard-window method, and by 20.1% relative to RSSI-MAP. The large error of unrestricted phase-only localization reflects the ambiguity and poor conditioning of the short collinear baselines; the RSSI-supported decision domain is therefore an essential component rather than a cosmetic preprocessing step.

A tag-stratified paired bootstrap with 5000 replicates gave 95% confidence intervals of 33.8% to 60.0% for the improvement over PRTFC, 76.1% to 82.4% over the original hard-window joint method, and 12.6% to 28.5% over RSSI-MAP.

**Figure 13:**
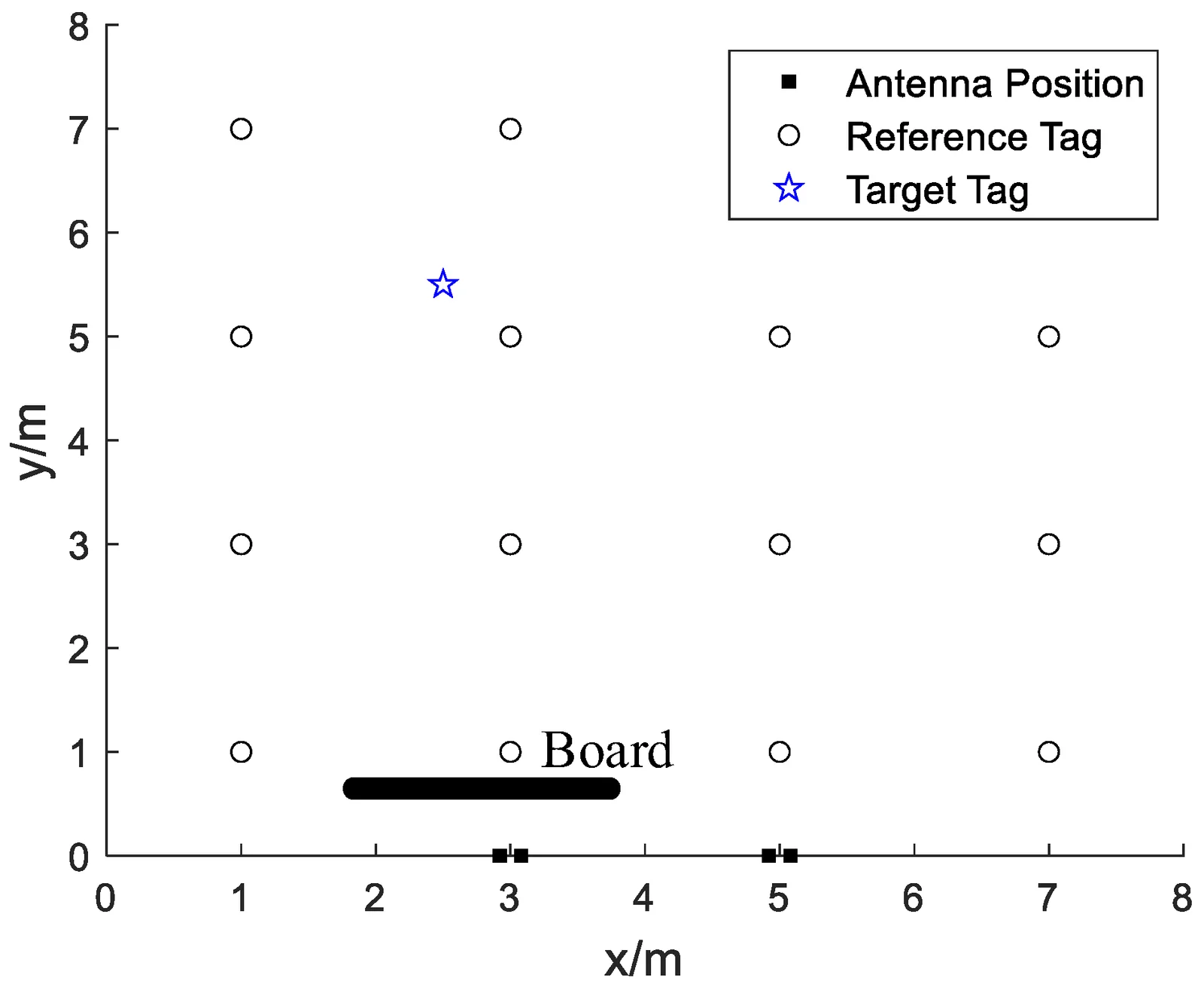
Positioning system deployment with an obstructing board.

For the mixed LOS/NLOS simulation, the same nine target coordinates and calibrated parameters were retained. Phase pair 1 was modeled using image antennas mirrored across the dominant wall x = 0, while phase pair 2 remained in LOS. Both the compensated and uncompensated formulations were evaluated on the same noise realizations, allowing the contribution of geometric reflection compensation to be isolated. The mixed-scene deployment is illustrated in Figure 13.

**Figure 14:**
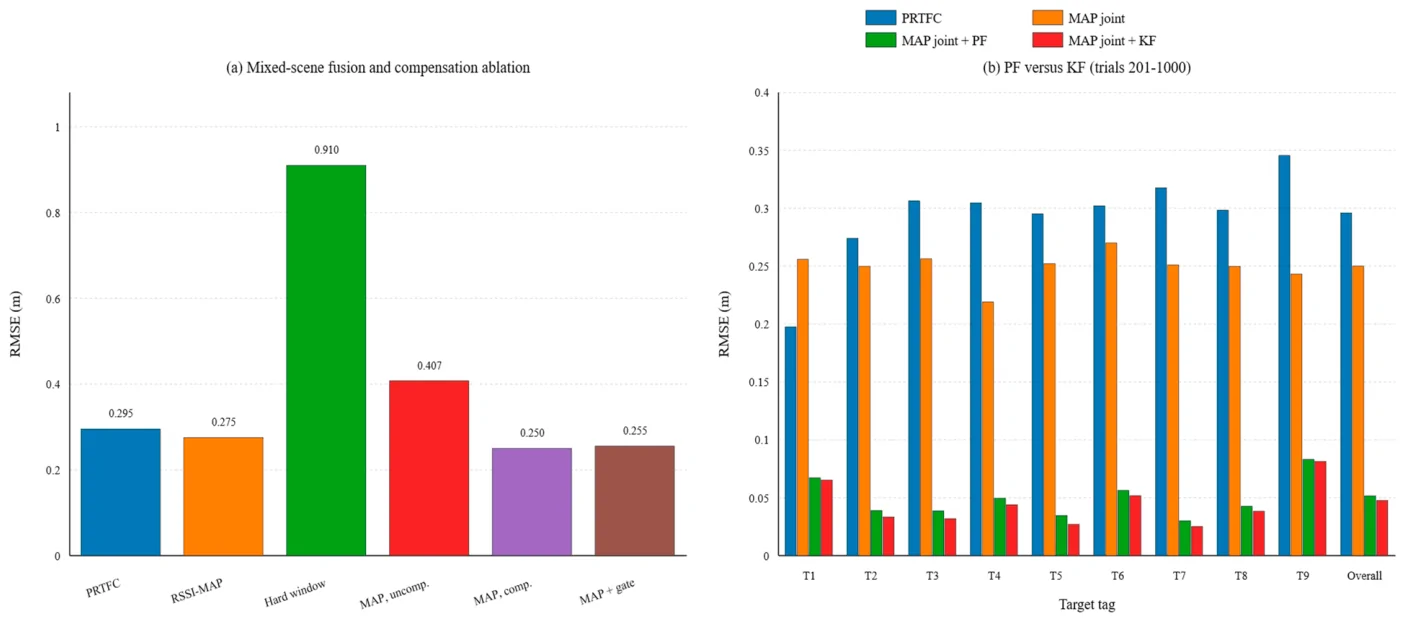
Mixed LOS/NLOS results: (**a**) fusion, reflection-compensation, and reliability-gate ablation; (**b**) per-tag comparison of PRTFC, the unfiltered compensated MAP joint estimator, PF, and KF over observations 201–1000 after burn-in.

Figure 14a reports the mixed LOS/NLOS ablation. The overall RMSEs of PRTFC, RSSI-MAP, the original compensated hard-window method, the uncompensated MAP joint method, and the compensated MAP joint method were 0.295, 0.275, 0.910, 0.407, and 0.250 m, respectively. Reflection compensation reduced the mixed-scene MAP error by 38.4% relative to the uncompensated formulation. The calibrated reliability gate changed the RMSE from 0.250 to 0.256 m and was therefore retained only as a negative ablation, not as part of the primary simulation estimator.

For the compensated MAP estimator, the paired-bootstrap 95% confidence interval for improvement was 23.6% to 48.8% relative to the uncompensated formulation and 6.5% to 12.4% relative to RSSI-MAP.

Figure 14b compares the temporal filters over observations 201–1000. The RMSEs of the unfiltered MAP estimator, PF-smoothed estimator, and KF-smoothed estimator were 0.250, 0.0516, and 0.0476 m, respectively. PF and KF reduced RMSE by 79.4% and 81.0% relative to the unfiltered estimator. KF had an 8.27% lower RMSE than PF in this stationary, approximately Gaussian sequence; this result should not be generalized to moving-target tracking without trajectory-based validation.

The 95% confidence intervals for the RMSE reductions relative to the unfiltered estimator were 74.6% to 84.0% for PF and 75.7% to 86.2% for KF. The paired estimate placed the KF reduction relative to PF between 4.64% and 16.18%.

The mean runtimes of PRTFC, standalone SD-PDOA, the original hard-window joint method, and the proposed MAP estimator were approximately 0.05, 0.82–0.87, 0.38, and 1.13 ms per estimate, respectively, in MATLAB R2024a on the test computer. The corresponding totals with PF and KF were approximately 1.16 and 1.14 ms. These algorithm-only values correspond to more than 850 estimates per second, but they are not an end-to-end reader update rate: reader dwell time and RF-switch scheduling were not preserved in the acquisition logs. We therefore report implementation-specific computation time without making a hardware-independent real-time claim.

Overall, the simulation confirms that normalized probabilistic fusion and candidate-wise ambiguity handling provide the main accuracy gain, while dominant-reflection compensation is beneficial when the environmental geometry is known. Both PF and KF reduce temporal variability; their relative advantage depends on the motion and noise model.

### 3.2. Real-World Experimental Validation

To further evaluate the performance and robustness of the proposed PRTFC-SDPDOA joint localization algorithm in practical applications, real-world experiments were conducted in an approximately 8 m x 8 m indoor positioning area.

The hardware platform consisted of an Impinj R420 reader operating at 920 MHz with a transmit power of 20 dBm. Each of the four reader ports was connected to an RF switch that time-division multiplexed one RSSI antenna and one carrier-phase antenna, providing four RSSI channels and four phase channels during each acquisition cycle. Sixteen real reference tags were deployed in a 4 × 4 grid to construct the RSSI fingerprint map. The surveyed phase-antenna centers were P1 (2.90, 0.04), P2 (3.10, 0.02), P3 (4.91, −0.03), and P4 (5.09, 0.01) m. Nine test locations were evaluated: (1.3, 1.8), (1.7, 3.9), (2.4, 5.9), (3.8, 1.4), (4.3, 4.2), (4.1, 6.7), (5.9, 2.1), (6.3, 5.8), and (6.7, 4.3) m.

**Figure 15:**
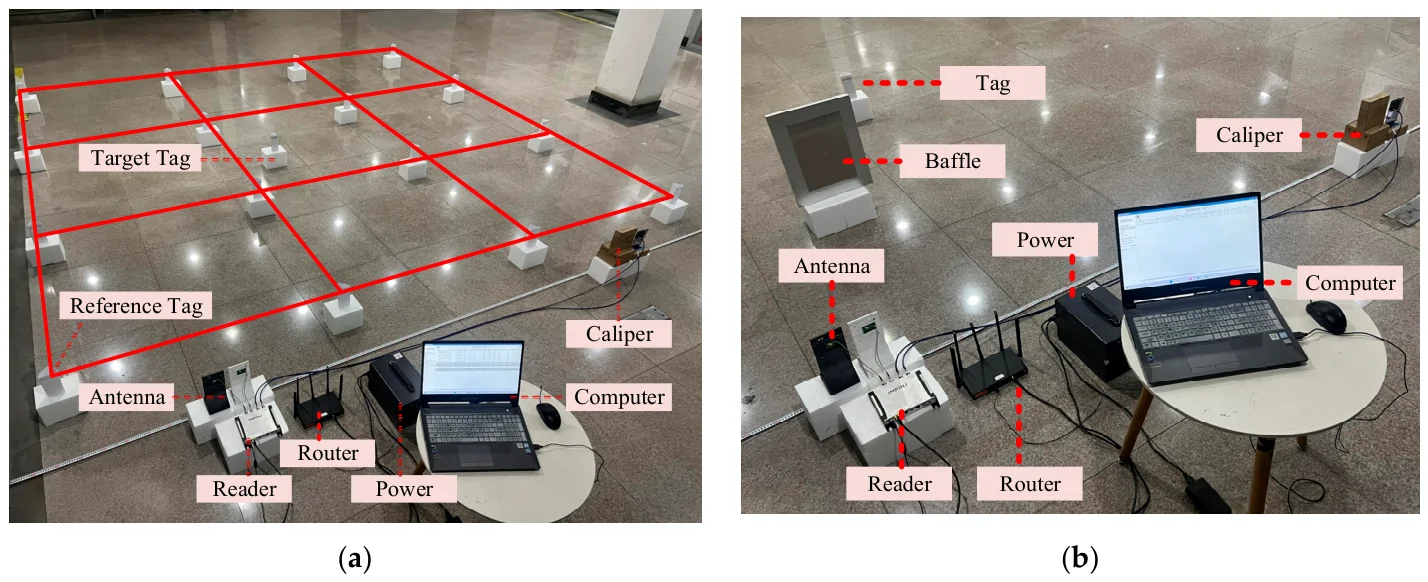
Representative photographs of the R420-based experimental platform: (**a**) the reader, antennas, host computer, and 4 × 4 reference-tag grid used for RSSI fingerprint construction under LOS conditions; (**b**) a representative obstruction arrangement for mixed LOS/NLOS measurements. The photographs illustrate the experimental platform, while the coordinates used in the analysis are specified in the text.

Two propagation conditions were evaluated. In LOS, the direct paths remained unobstructed. In the mixed condition, a metal cabinet and a movable absorber were placed between the target region and phase pair P12. Five hundred cycles were scheduled at each location and condition. The acquisition software retained the first successful read for each channel, ignored duplicates, and did not interpolate unresolved observations. Complete cycles without flagged outliers were used, yielding 4361 LOS and 4061 mixed-scene cycles. Antenna-specific circular calibration offsets were removed before forming the phase differences. The RSSI model standard deviation was fixed at 1 dB and the decision-domain threshold at 8; the candidate grid spacing was 0.05 m. Because the exact reflecting-surface survey was not retained for this campaign, the known obstructed pair P12 was assigned zero phase reliability in the mixed-scene joint estimator, while P34 remained active. No true test coordinate was used for this decision or for estimator initialization. PF and KF used the same fixed parameters as in the simulation. Per-location RMSE was calculated from all retained cycles and then averaged over the nine locations. Representative photographs of the experimental platform are shown in Figure 15.

#### 3.2.1. Localization Performance Under LOS Conditions

**Figure 16:**
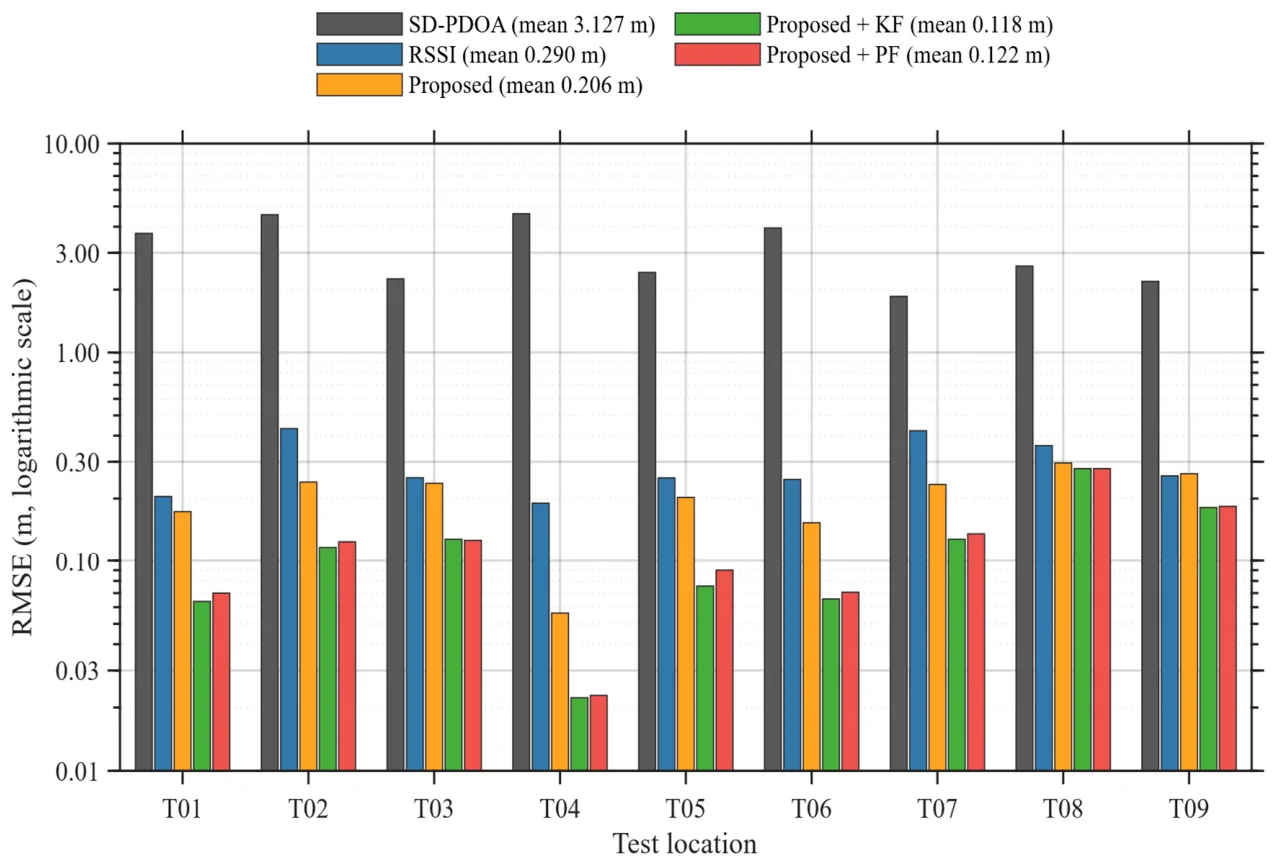
Nine-position experimental RMSE under LOS conditions. The bars show standalone SD-PDOA, RSSI fingerprint localization, the unfiltered proposed joint estimator, and its KF- and PF-filtered outputs. The ordinate is logarithmic; legend values are averages over the nine test locations.

Figure 16 compares five LOS estimators at the nine measured locations: unrestricted standalone SD-PDOA, RSSI fingerprint localization, the unfiltered proposed joint estimator, and the same joint estimates processed by KF or PF. A logarithmic ordinate is used because the unrestricted phase-only solution spans a substantially larger error range than the RSSI-supported estimators.

The mean point-wise RMSEs were 3.127 m for standalone SD-PDOA, 0.290 m for RSSI, 0.206 m for the unfiltered proposed estimator, 0.118 m after KF, and 0.122 m after PF. The unfiltered joint method improved on RSSI at eight of the nine locations, while both filters improved on RSSI at all nine locations. Relative to RSSI, PF reduced the average RMSE by 57.9%. KF was approximately 4.0% lower than PF in this stationary LOS experiment, although the two results were close in absolute terms.

The tag-stratified 95% confidence intervals for mean point-wise RMSE were 2.500–3.785 m for standalone SD-PDOA, 0.237–0.348 m for RSSI, 0.159–0.246 m for the unfiltered joint estimator, 0.075–0.169 m for KF, and 0.081–0.173 m for PF.

#### 3.2.2. Localization Performance Under Mixed LOS/NLOS Conditions

For the mixed LOS/NLOS experiment, the metal cabinet and movable absorber obstructed phase pair P12 while P34 remained available. Standalone SD-PDOA continued to use both measured pairs and therefore quantifies the consequence of unrestricted phase localization under the obstruction. The proposed estimator used the RSSI-supported domain and the reliable P34 phase observation, followed by the same fixed KF and PF configurations.

The mean point-wise RMSEs in the mixed scene were 3.608 m for standalone SD-PDOA, 0.808 m for RSSI, 0.685 m for the unfiltered proposed estimator, 0.424 m after KF, and 0.457 m after PF. The unfiltered joint estimator improved on RSSI at seven of nine locations, and both filtered outputs improved on RSSI at all nine. PF reduced the average RMSE by 43.5% relative to RSSI, whereas KF was 7.7% lower than PF. Thus, the real measurements confirm the value of RSSI-constrained phase fusion and temporal smoothing, but they do not support claiming that PF is universally superior to KF.

The corresponding tag-stratified 95% confidence intervals were 3.024–4.190 m for standalone SD-PDOA, 0.628–1.000 m for RSSI, 0.559–0.843 m for the unfiltered joint estimator, 0.309–0.551 m for KF, and 0.311–0.619 m for PF.

## 4. Discussion

The revised results separate the contributions of coarse localization, phase refinement, reflection compensation, and temporal filtering. In simulation, replacing the original hard decision window with a connected RSSI likelihood region and normalized MAP fusion reduced the LOS RMSE below both PRTFC and dense RSSI-MAP. The mixed-scene ablation further showed that a correct dominant-reflection model materially improves the joint estimate. In the physical experiment, RSSI constrained the phase ambiguity and the reliable phase pair refined the solution, after which both PF and KF reduced the variability of repeated static-tag observations.

**Figure 17:**
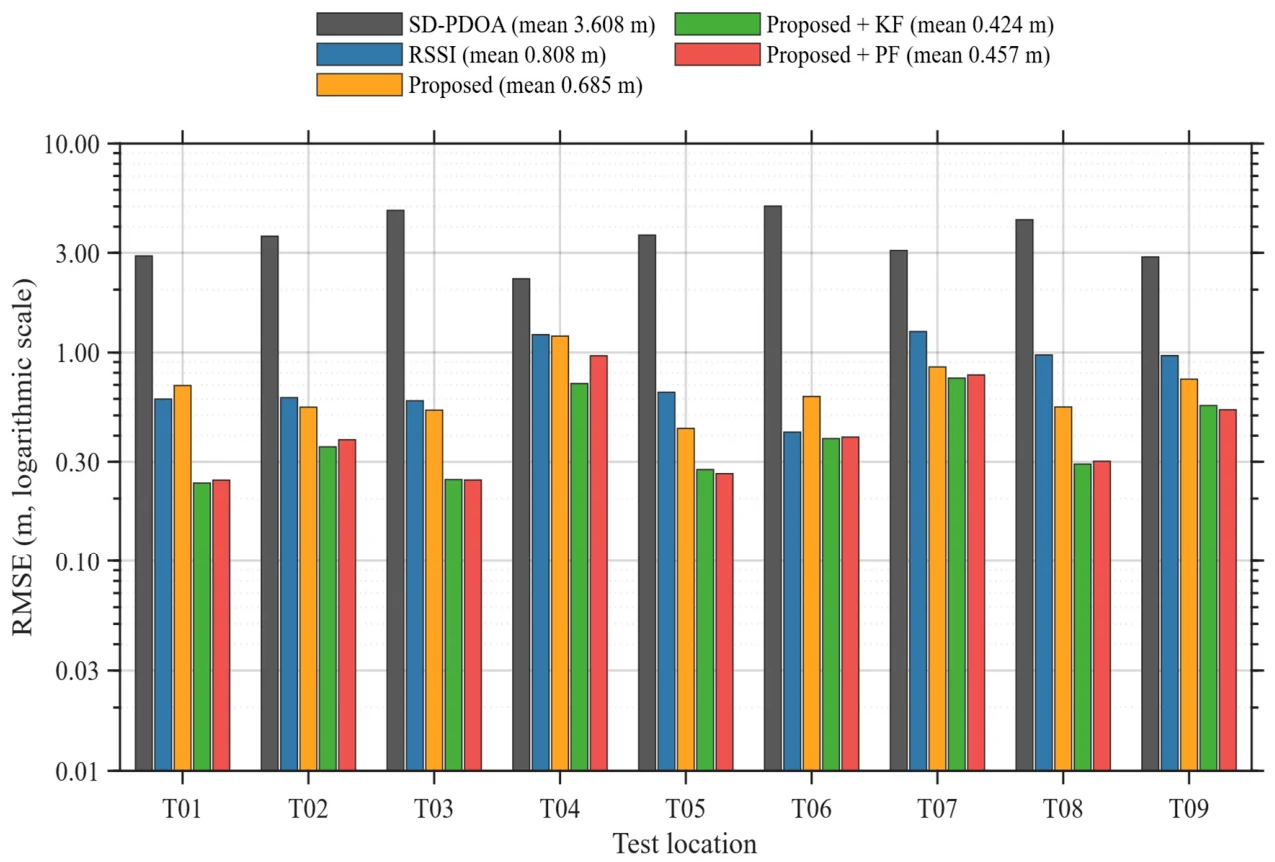
Nine-position experimental RMSE under mixed LOS/NLOS conditions. Phase pair P12 was deliberately obstructed; the proposed joint estimator used the RSSI-supported domain and reliable pair P34. The ordinate is logarithmic; legend values are averages over the nine test locations.

The filtering comparison also qualifies the original PF claim. KF was slightly more accurate in both measured scenarios because the tags were stationary and the residual fluctuations were sufficiently close to the random-walk/Gaussian assumptions. PF remains useful for nonlinear or multimodal tracking, but demonstrating that advantage requires moving trajectories and cannot be inferred from repeated measurements at fixed points. In addition, the physical mixed-scene records did not preserve an independent high-precision survey of the reflecting surface; consequently, Figure 17 validates known-link rejection and joint localization, whereas the quantitative reflection-compensation claim is supported by the controlled simulation ablation in Figure 14.

Robustness to map and obstacle inaccuracies remains an important limitation. The image-antenna transformation depends on the position and orientation of the dominant reflecting surface. The RSSI likelihood region limits the effect of moderate phase-model mismatch, but it cannot replace an accurate environmental map. The experimental results include ordinary antenna-placement and obstacle-placement uncertainty, yet the absence of an independent survey log means that they should not be interpreted as establishing a specific centimeter-level tolerance to map errors.

The physical validation is limited to one approximately 8 m × 8 m indoor site, nine stationary test positions, two propagation configurations, and one reader/antenna layout. It does not establish robustness across buildings, obstacle materials and sizes, antenna-array geometries, or tag orientations. The available photographs document the metal cabinet and movable absorber, but contemporaneous records of their exact dimensions and material composition and a high-precision reflector survey were not retained. We therefore avoid a material-specific robustness claim.

Deployment after an environmental change requires recalibration at the layer affected by that change. The RSSI fingerprint map should be refreshed after moving antennas or reference tags or after material changes to room geometry and furnishings. Antenna phase offsets should be recalibrated after changes to cables, switches, antennas, or carrier settings. The image-antenna model should be updated when the dominant reflector moves or rotates. Timing changes alone do not require a new spatial calibration if the hardware configuration and validation-tag residuals remain stable. Multi-room tests, controlled material and obstacle-position sweeps, tag-orientation trials, and moving trajectories are left for future work.

## 5. Conclusions

This study developed a passive RFID localization framework combining an RSSI fingerprint likelihood, candidate-wise SD-PDOA ambiguity resolution, normalized probabilistic fusion, dominant-reflection compensation, and temporal filtering. The independent simulation produced RMSEs of 0.098 m in LOS and 0.250 m in the mixed scene, and reflection compensation reduced the mixed-scene joint RMSE by 38.4% relative to the uncompensated formulation. Across nine physical test locations, PF achieved mean point-wise RMSEs of 0.122 m in LOS and 0.457 m under mixed propagation; KF achieved 0.118 and 0.424 m. The measurements therefore support the core conclusion that RSSI-constrained phase fusion and temporal filtering improve passive-tag localization, while also showing that KF is the more suitable filter for the stationary, approximately Gaussian sequences tested here. Further work should evaluate preserved reflecting-surface surveys and moving-target trajectories.

## Data Availability

The processed cycle-level R420 measurements, calibration summaries, simulation outputs, and custom scripts supporting the findings of this study are provided in the Supplementary Materials. The raw lower-level binary logs generated by the reader were not retained.

## Supplementary Materials

The following supporting information can be downloaded at: https://www.mdpi.com/article/10.3390/s26196314/s1, processed cycle-level R420 measurements, calibration summaries, simulation outputs, and custom scripts supporting the results presented in this study.

## Abbreviations

The following abbreviations are used in this manuscript:

RFID	Radio Frequency Identification
RSSI	Received Signal Strength Indication
PDOA	Phase Difference of Arrival
LOS	Line-of-Sight
NLOS	Non-Line-of-Sight
SD-PDOA	Spatial Domain-Phase Difference of Arrival
PRTFC	Pseudo-Reference Tag Feature Comparison
PF	Particle Filter
UWB	Ultra-wideband
UHF	Ultra-High Frequency
TDOA	Time Difference of Arrival
AOA	Angle of Arrival
