# Supplementary material for: A Passive Localization Algorithm That Integrates RSSI and PDOA Location Observations in a Mixed Scene
Source: Sensors (Basel). 2026 Oct 6;26(19):6314. doi: 10.3390/s26196314 (PMC13667580; doi:10.3390/s26196314)
Supplement: Supplementary file 1 [file sensors-26-06314-s001.zip › Supplementary_Information.pdf]

Supplementary Materials

Scope of the supplementary package

This package provides the data, code, parameters, figures, and audit outputs used for the revised simulation and nine-position R420 experiment. It supports Figures 11, 12, 14, 16, and 17 and the reported ablation and PF–KF comparisons. The experiment files originate from an Impinj R420 reader and an upper-computer acquisition program. The reader's lower-level binary log was not retained. The most upstream retained record is the row-wise CSV written by the acquisition program. MAT compatibility files were prepared after anomaly screening. Missing antenna observations were not filled with zeros or interpolated values. Spatial interpolation was used only to form the RSSI fingerprint map from reference-tag measurements.

S1. Package organization

Directory	Contents	Use
01_Figure11_PRTFC_LOS_Simulation	Figure 11, LOS PRTFC results and supporting summaries	Locate and inspect Figure 11
02_Figure12_LOS_Three_Algorithm_Comparison	Figure 12 and matched LOS method-comparison tables	Locate and inspect Figure 12
03_Figure14_Mixed_LOS_NLOS_Simulation	Figure 14, Figure S1, gate ablation and calibration tables	Locate and inspect Figure 14 and S1
04_Figure16_Nine_Position_LOS_Experiment	Figure 16 and nine-position LOS metrics	Locate and inspect Figure 16
05_Figure17_Nine_Position_Mixed_LOS_NLOS_Experiment	Figure 17 and nine-position mixed-scene metrics	Locate and inspect Figure 17
06_Nine_Position_Experimental_Data_Calibration_and_Results	R420 source data, calibration inputs and reported results	Audit experimental data provenance
07_Reproduction_Code_Parameters_and_Environment	Portable MATLAB and Python reproduction code	Rerun simulations and experiment analysis
08_Complete_Results_Trial_Data_and_Verification	Complete trial outputs, development repeat and SHA-256 records	Audit detailed outputs and package integrity

Table S1 Package organization

S2. Simulation design and reproducibility

The formal simulation uses a common-random-number design for paired method comparisons. Calibration and evaluation use separate fixed seeds. Each test position is evaluated with 1000 observations after parameters are fixed. The mixed scene models a dominant reflection for phase pair P12 and retains pair P34 as LOS.

Item	Setting
Deployment area	8 m × 8 m
RSSI antennas	(0,0), (0,8), (8,0), (8,8) m
Phase antennas	(2.92,0), (3.08,0), (4.92,0), (5.08,0) m
Carrier frequency and power	920 MHz and 20 dBm
Reference tags	16 physical tags at $x,y \in \{1,3,5,7\}$ m
Fingerprint grid	0.5 m spacing
LOS noise	RSSI 0.2 dB; phase 0.1 rad
Mixed-scene noise	RSSI 0.5 dB; phase 0.3 rad
Evaluation	9 test positions; 1000 observations per position

Table S2 Formal simulation settings

S3. Experimental acquisition and processing

Reader ports ANT1–ANT4 were connected to R1/P1, R2/P2, R3/P3, and R4/P4, respectively. An RF switch behind each port alternated between the RSSI and phase antennas. Only one antenna path was connected to a reader port at a time. The software recorded the eight observations sequentially within each cycle.

Item	Recorded setting
Reader	Impinj R420
Carrier frequency	920 MHz
Transmit power	20 dBm
RSSI antennas	Four antennas, R1–R4
Phase antennas	P1 (2.90,0.04), P2 (3.10,0.02), P3 (4.91,-0.03), P4 (5.09,0.01) m
Reference tags	16 tags in a 4 × 4 layout
Test positions	Nine positions, T01–T09
Scenes	LOS and mixed LOS/NLOS
Mixed-scene obstruction	Metal cabinet and movable absorber affecting P12; P34 retained
Planned observations	500 cycles per position and scene

Table S3 R420 experiment settings

S4. Data cleaning and sample accounting

Records were grouped by scene, position, cycle, and read index. The first successful OK or RETRY_OK record was retained for each channel. DUPLICATE records were ignored. A channel remained missing when no successful record was available after retries. The main analysis retained cycles with CompleteFourAntennaCycle equal to 1 and OutlierCount equal to 0.

Scene	Planned cycles	Complete cycles	Complete and non-outlier cycles	Used in analysis
LOS	4500	4438	4361	4361
Mixed LOS/NLOS	4500	4258	4061	4061
Total	9000	8696	8422	8422

Table S4 Experimental sample accounting

S5. Localization and filtering parameters

Parameter	Value
Real-data search-grid spacing	0.05 m
RSSI likelihood standard deviation	1.0 dB
RSSI support threshold	ΔNLL = 8
PF particle count	500
PF process standard deviation	0.01 m
PF measurement standard deviation	0.30 m
PF initial standard deviation	0.30 m
PF resampling rule	Systematic resampling when ESS < 0.5K
KF process variance	1×10^{-5} m^2
KF measurement variance	0.0225 m^2

Table S5 Parameters fixed before the nine-position evaluation

S6. Experimental localization results

Scene	Method	Cycles	Mean point-wise RMSE m	95 percent CI m	Pooled P90 error m
LOS	SD-PDOA	4361	3.127	2.500–3.785	5.657
LOS	RSSI	4361	0.290	0.237–0.348	0.461
LOS	Proposed	4361	0.206	0.159–0.246	0.354
LOS	Proposed + PF	4361	0.122	0.081–0.173	0.248
LOS	Proposed + KF	4361	0.118	0.075–0.169	0.251
Mixed LOS/NLOS	SD-PDOA	4061	3.608	3.024–4.190	5.657
Mixed LOS/NLOS	RSSI	4061	0.808	0.628–1.000	1.320
Mixed LOS/NLOS	Proposed	4061	0.685	0.559–0.843	1.051
Mixed LOS/NLOS	Proposed + PF	4061	0.457	0.311–0.619	0.783
Mixed LOS/NLOS	Proposed + KF	4061	0.424	0.309–0.551	0.735

Table S6 Nine-position experimental localization results

For the stationary repeated-observation sequences, both temporal filters reduced the unfiltered joint-estimator RMSE. The KF was slightly more accurate than the PF under the fixed approximately Gaussian measurement model. This result does not establish superiority for moving-tag tracking.

S7 Simulation comparison and ablation

Scene	Paired comparison	RMSE reduction percent	95 percent CI
LOS	MAP joint versus PRTFC	49.78	33.77 to 60.05
LOS	MAP joint versus Hard-window joint	80.21	76.11 to 82.38
LOS	MAP joint versus RSSI-MAP	20.07	12.64 to 28.53
Mixed	MAP joint compensated versus MAP joint uncompensated	38.42	23.58 to 48.83
Mixed	MAP joint compensated versus RSSI-MAP	9.38	6.50 to 12.41
Mixed	MAP joint compensated + PF versus MAP joint compensated	79.38	74.57 to 84.00
Mixed	MAP joint compensated + KF versus MAP joint compensated	80.95	75.70 to 86.22
Mixed	MAP joint compensated + PF versus MAP joint compensated + KF (negative favors KF)	-8.27	-16.18 to -4.64

Table S7 Paired simulation comparisons

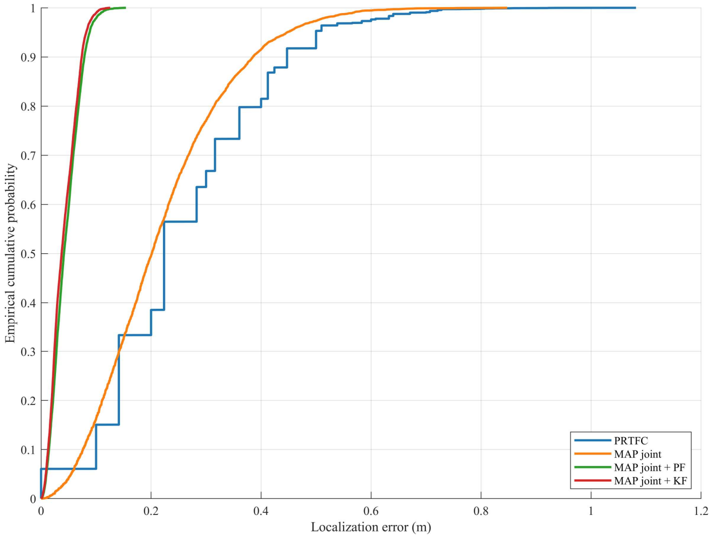

Figure S1 Empirical localization-error distributions in the formal mixed-scene simulation.

S8. Reproduction instructions

Formal simulation

- Open MATLAB in 07_Reproduction_Code_Parameters_and_Environment/Joint_Simulation_Code and add code/joint to the path.
- Run run_joint_rerun(fullfile(pwd,'reproduced'),NaN,NaN,20260924).
- Run create_joint_publication_outputs(fullfile(pwd,'reproduced')).

R420 experiment

- Install Python 3.12 with NumPy and pandas.
- Run python run_nine_point_analysis.py from 07_Reproduction_Code_Parameters_and_Environment/Nine_Position_Experiment_Code.
- Run make_real_figures.m from 07_Reproduction_Code_Parameters_and_Environment/Nine_Position_Experiment_Code to regenerate Figures 16 and 17.

The simulation seed is fixed, so numerical estimates should agree apart from runtime measurements. The experimental analysis is deterministic except for the PF and bootstrap confidence intervals, which use the fixed seed reported above.

S9. Known limits and interpretation

- The lower-level reader binary log is unavailable; the row-wise acquisition-software CSV is the earliest retained record.
- The original anomaly-detection threshold cannot be recovered uniquely. The distributed dataset retains cycle-level flags, and the main analysis applies the stated reproducible exclusion rule.
- Exact reflector survey coordinates were not retained. The real mixed-scene analysis therefore disables phase pair P12 and retains P34 rather than fitting an unverified reflector model.
- PF and KF results describe stationary repeated observations and should not be interpreted as moving-target tracking accuracy.
